# Auditory processing in children: Role of working memory and lexical ability in auditory closure

**DOI:** 10.1371/journal.pone.0240534

**Published:** 2020-11-04

**Authors:** Naveen K. Nagaraj, Beula M. Magimairaj

**Affiliations:** Department of Communicative Disorders and Deaf Education, Cognitive Hearing Science Lab, Utah State University, Logan, Utah, United States of America; University of Florida, UNITED STATES

## Abstract

We examined the relationship between cognitive-linguistic mechanisms and auditory closure ability in children. Sixty-seven school-age children recognized isolated words and keywords in sentences that were interrupted at a rate of 2.5 Hz and 5 Hz. In essence, children were given only 50% of speech information and asked to repeat the complete word or sentence. Children’s working memory capacity (WMC), attention, lexical knowledge, and retrieval from long-term memory (LTM) abilities were also measured to model their role in auditory closure ability. Overall, recognition of monosyllabic words and lexically easy multisyllabic words was significantly better at 2.5 Hz interruption rate than 5 Hz. Recognition of lexically hard multisyllabic words and keywords in sentences was better at 5 Hz relative to 2.5 Hz. Based on the best fit generalized “logistic” linear mixed effects models, there was a significant interaction between WMC and lexical difficulty of words. WMC was positively related only to recognition of lexically easy words. Lexical knowledge was found to be crucial for recognition of words and sentences, regardless of interruption rate. In addition, LTM retrieval ability was significantly associated with sentence recognition. These results suggest that lexical knowledge and the ability to retrieve information from LTM is crucial for children’s speech recognition in adverse listening situations. Study findings make a compelling case for the assessment and intervention of lexical knowledge and retrieval abilities in children with listening difficulties.

## Introduction

Listening in everyday environments can be challenging for children when optimal listening conditions are frequently disrupted. Background noise levels in classrooms often exceed the minimum recommended standards [1]. Children who are diagnosed to have developmental disorders such as Developmental Language Disorder (DLD), Auditory Processing Disorder (APD), Dyslexia, and Attention-Deficit Hyperactivity Disorder (ADD/ADHD) are especially at a greater disadvantage while listening in noise [2–5]. Listening in noisy learning environments can lead to greater cognitive effort and can potentially interfere with academic performance. To maximize the benefit from learning experiences in noisy environments, the ability to fill-in missing sensory information is crucial for children.

One way in which listeners cope when speech is masked by noise is through “auditory closure” (also known as perceptual restoration), wherein listeners tap into cognitive and linguistic resources to extract meaning from partial auditory information [6–9]. Listeners temporally integrate partial “glimpses” of spectro-temporal speech cues with the help of top-down restoration mechanisms [10,11]. In adults, large individual variability has been reported in how language and cognitive mechanisms are recruited during auditory closure tasks [12,13]. By 5 years of age, children demonstrate perceptual restoration ability [14], however, they are not as effective as adults in reconstructing missing speech [14–17]. This is potentially due to significant differences in the way children and adults deploy top-down mechanisms to interpret disrupted auditory input. Furthermore, auditory closure is challenging for a subset of normal hearing children, who are diagnosed to have APD or DLD.

In the upper mid-western United States, the prevalence of DLD in kindergarten children is estimated to be 7.4% [18]. Children with DLD have language comprehension and expression problems in the absence of other conditions such as hearing loss, intellectual disability, or frank neurological deficits. Children with DLD may miss incoming speech information due to memory deficits, slower processing speed, noisy environments, distractions, attention deficits, or low phonetic substance [19–26]. These factors have been identified as potential areas of weakness in children with DLD and children diagnosed with ADD/ADHD [27]. Whereas the actual prevalence of APD is unknown [28], estimates suggest that up to 5% of school-age children have APD [29]. In fact, auditory processing deficits may co-occur in children with DLD, dyslexia, or ADHD [27,30,31].

When listening to degraded speech [32,33], auditory closure, the brain’s ability to *fill-in* missing information kicks in to facilitate listening comprehension [12,34]. For example, listeners’ ability to integrate pieces of information over time (temporal integration) and ability to use perceptual skills such as timing, intonation, and loudness, may aid restoration of missing information [35]. Factors such as contextual information, vocabulary knowledge, lexical effects including word frequency and phonemic neighborhood density, and phonological representation/knowledge are also critical [7,8,36,37]. Cognitive mechanisms such as attention and working memory, however, have received little attention with reference to auditory closure [38,39].

Auditory closure has conventionally been evaluated in auditory processing test batteries using monoaural low-redundancy speech tasks, low-pass filtered words, time-compressed words, and speech-perception-in-noise tests. These tasks represent listening in degraded or low context/low redundancy conditions. Linguistic closure is also measured as part of language tests in tasks such as sentence completion or completion of word endings (i.e., bound morphemes). Other methods to measure closure include lexical/time gating and recognition of/judgments about auditory stimuli based on external redundancy (transparency or context information provided by the stimuli). Another paradigm that closely measures the ability to fill-in *missing* information, is the interrupted speech perception task. This task has been used in several studies to assess perceptual restoration of missing speech information in adults. In the interrupted speech perception task, speech segments are removed thereby leaving silent gaps [9,40–42]. Alternatively, the silent gaps are replaced with noise thus forcing listeners to recognize words or sentences with limited speech information [43]. The interrupted speech perception task can be used to study how individuals integrate glimpses of controlled speech input with the aid of cognitive-linguistic mechanisms to restore missing speech information. Whereas the interrupted speech perception task serves the purpose of measuring auditory closure similar to other paradigms listed above, it allows more systematic control of the total duration/amount of information provided/missing. The difference in performance between silent-gap and noise-filled conditions represents perceptual restoration ability.

Newman [14] measured perceptual restoration ability in school-age children and adults to examine whether children showed improved speech perception similar to adults (when missing speech intervals were filled with noise relative to the silent condition). High predictability sentences from a speech perception in noise test were used in silence and noise-filled conditions, respectively [44]. Interruption interval rates (alternating speech and gap durations) were 250 ms, 200 ms, 150 ms, and 100 ms. Adults were asked to type the sentences they heard whereas children were asked to repeat the sentences they heard. Overall, accuracy was greater in adults than in children with both silent and noise-filled interrupted speech conditions. However, perceptual restoration ability in children (i.e., difference in performance between silent and noise-filled condition) was comparable to adults. This suggests that even though children have smaller lexical networks than adults, they did demonstrate the ability to deploy linguistic knowledge to interpret interrupted speech. An important next question is determining the factors that underlie individual differences in children’s auditory ability.

Studies in adults suggest that vocabulary, and acoustic and phonological processing influence auditory closure [12,13]. However, there are limited studies on school-age children’s auditory closure ability that also examine constraining cognitive-linguistic factors. The majority of existing studies have focused on lexical effects in spoken word or sentence recognition in degraded conditions by normal hearing children and children with hearing loss [16,36,45]. Researchers have demonstrated that children are significantly more accurate at recognizing words which are high in frequency of occurrence and have a sparse phonemic neighborhood, i.e., phonemically dissimilar neighbors than low frequency words from a dense phonemic neighborhood with many phonemically similar words [36,45,46]. The weightage of lexical neighborhood density is reported to be greater than word frequency for speech recognition in children [45]. Similar to lexical effects, high syntactic context and high predictability results in better recognition of sentences by children than sentences with low syntactic context and low predictability [36,47].

Using a spoken word recognition paradigm in noise, Fort et al., [48] showed that children were significantly better at detecting a missing phoneme in words than in pseudo-words. This suggested that lexical knowledge biased speech perception. Furthermore, children showed better phoneme detection in audio-visual compared to auditory only condition and this modality advantage increased with age from 6 years. An interesting additional finding was that there was no interaction between lexical context and modality, which suggested that when listening in noise, lexical factors did not significantly advantage speech perception in the audio-visual condition. Recently, Walker and colleagues [49] examined the role of working memory and vocabulary on time-gated word recognition in children with hearing loss. They found that lexical knowledge, not working memory capacity, mediated the relationship between audibility and recognition of time-gated words. Two studies in school-age typically developing children have examined perception of interrupted melodies (tunes of rhymes) and spoken word recognition with missing phoneme information [17]. Studies in younger children have also suggested that children’s speech perception is more vulnerable to interruptions than adults. That is, they show less perceptual restoration than adults [14,16]. Studies in children with and without learning disorders have used the forward gating paradigm to examine word identification [50,51]. The general findings from these studies suggested that children with and without learning disabilities/developmental language disorder were comparable in acoustic-phonetic analysis/auditory closure ability and the mean duration needed for word identification. Receptive vocabulary was associated with word identification in children with learning disability [50].

Systematic study of factors influencing individual variability in auditory closure has clinical significance because some children have processing capacity limitations which may lead to information loss and hence the need to accomplish auditory closure. Auditory processing difficulties may be associated with or be a consequence of language impairment [52] and auditory closure is often targeted as part of language intervention. The current study in school-age children across a broad cognitive range is foundational to future studies in children with processing limitations such as short-term memory or working memory deficits, slower processing speed, listening difficulty in noise, attention deficits, or language processing deficits.

To systematically study children’s ability to reconstruct missing speech by integrating available speech information, we used the interrupted speech perception paradigm [32]. The specific aim was to examine the contribution of cognitive-linguistic mechanisms on perceptual restoration of missing speech. Interrupted speech with filler noise sounds continuous, and this illusion of perceived speech continuity is expected to activate a larger lexical network in listeners’ long-term memory [53]. The main prediction was that children’s auditory closure ability would be significantly influenced by lexical knowledge and their ability to accurately retrieve activated words from LTM. Based on previous studies in adults [13] and children [49] we predicted that cognitive mechanisms such as working memory and attention control may not be directly related to auditory closure ability in children.

## Method

### Participants

Children 8- to 11 years old, were invited to participate in an IRB approved project at the University of Central Arkansas. The primary caregiver and child provided informed consent prior to participation. The primary caregiver also completed a questionnaire about the child’s developmental history. Exclusionary criteria were hearing loss, intellectual disabilities, stuttering, autism, seizure disorder, or frank developmental or acquired neurological disorders. All qualifying children were administered the same tasks in fixed order over two sessions. The order of conditions within the speech perception task was counterbalanced across participants. At each visit, the caregiver received a gift card and a toy prize was given to the child. A free hearing and language screening report was also provided. Based on previous research [54] and supporting literature [55], typically developing children make significant transitions in certain cognitive abilities such as attention switching, a critical predictor of working memory performance, around the age of 7 years. Eight-year-olds show stable performance, with 12-year-olds performing at ceiling on cognitive and auditory processing tasks designed for this age range. Given this developmental trend, 8- to 11-year-olds were recruited for this study.

Sixty-seven school-age children participated. Majority of the children were typically developing. Three children had individualized educational plans and four children had accommodation (504) plans such as extended time or tutoring help. The complete sample was used for data analysis and reporting of results as the children represented a continuum of individual differences in language and cognitive abilities. Importantly, all participating children demonstrated normal-range hearing, articulation, and non-verbal IQ based on screening. Children who passed screening were administered multiple language, cognitive, and interrupted speech perception tasks. All auditory stimuli were presented under headphones (Senheisser HD280 Pro).

### Tasks

#### Auditory closure: Interrupted speech perception task

**Fig 1:**
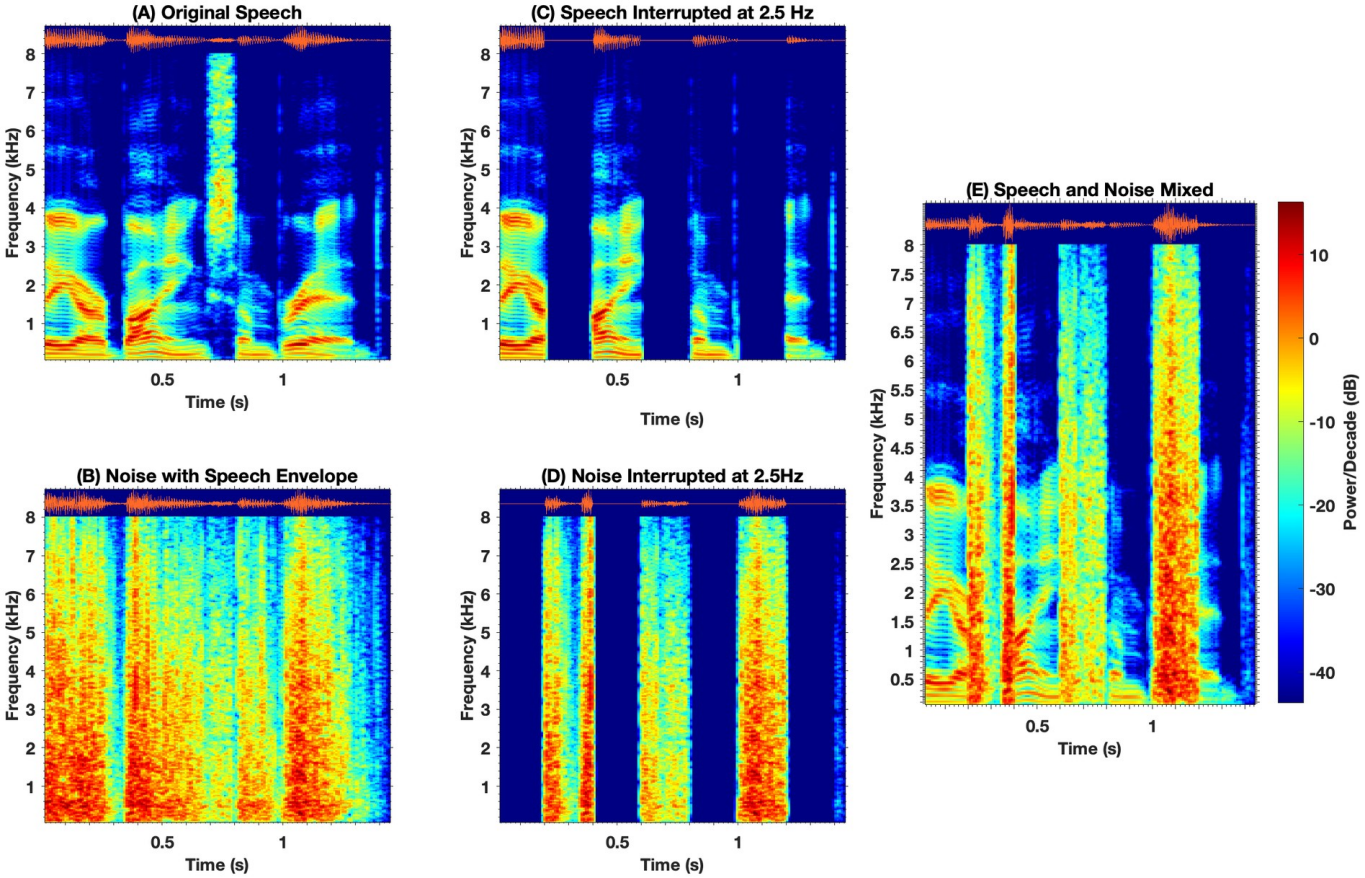
Waveform and spectrogram illustration of clean speech “*They are buying some bread*” (A), noise modulated using the original speech envelope (B), interrupted speech at 2.5Hz with three 200ms glimpses of clean speech per second (C), interrupted noise (D) and combined interrupted sentence with alternating segments of speech and noise (E).

*Stimuli generation*. Stimuli consisted of words from the Lexical neighborhood test [56] and sentences from the Bamford Kowal Bench Speech-in-Noise Test [57]. These were used to create interrupted stimuli. Interrupted speech with filler noise was created offline using MATLAB as illustrated in Fig 1. Original speech stimuli (Fig 1A) were first processed using Chimeric software [58] to extract the single channel broad band (80 to 8000Hz) temporal envelope. The extracted temporal envelope was then used to modulate the amplitude of speech shaped noise (Fig 1B). Speech shaped noise (SSN) was generated to have the same long-term power spectrum density as that of the BKB sentences and LNT words. Original speech stimuli (Fig 1A) were then gated with 50% duty cycle square wave at 2.5Hz or 5 Hz respectively to create silent gated speech stimuli (Fig 1C). The selection of interruption rates was based on a previous study in adults (13) and pilot data in children. Using the inverted square wave, envelope modulated SSN (Fig 1B) was gated to create an interrupted SSN (Fig 1D). To minimize distortion associated with abrupt gating of speech and noise, 5-ms raised cosine ramps were applied to the onset and offset to each cycle of the square wave. Finally, interrupted speech and noise were added to create the final stimuli (Fig 1E). The amplitude of filler speech shaped noise (SSN) was 8 dB higher than the replaced speech segments. The reason for filling the silent interval with envelope matched noise was based on evidence that the strength of perceptual restoration is lower when using stochastic white noise [59]. The interrupted speech stimuli always started with a clear speech segment.

*Procedure*. Children were asked to repeat the word or sentence presented via headphones. The dependent variable was keywords scored as correct or incorrect at two rates 2.5 Hz and 5 Hz. Perception of isolated monosyllabic words included 20 lexically easy and 20 lexically hard words for each rate. Multisyllabic words included 10 lexically easy and 10 lexically hard words at each rate. Perception of sentences included forty sentences per condition with 124 keywords scored per condition.

#### Working memory capacity measures

*Phonological STM*. The nonword repetition measure developed by Dollaghan and Campbell [60] was used. This measure was designed as a knowledge and background independent measure of language ability that particularly indexed children’s phonological STM (auditory-phonological processing, memory, and output organization). Children were presented a list of nonwords (e.g., /*naib*/) and were asked to repeat each nonword right after it was presented. The length of a nonword ranged from 1 to 4 syllables with four nonwords at each length. Accuracy of each consonant or vowel (diphthong) was scored as 0 or 1. The maximum possible score was 96.

*Working memory span*. This working memory measure was based on the complex span paradigm with processing and storage components [61]. Stimuli were computer-paced and practice for each task component preceded the test items. First, children saw a single-digit number on the screen followed by a next screen with two red squares on the top portion of the screen (i.e., small-small; big-big; small-big; or big-small randomly presented). The child is asked to judge if the two squares they saw were same or different and provide their answer by touching a box labeled “Same” or “Different” on the lower half of the screen. On practice trials it was confirmed that each child could read the words same/different. After a same-different judgment, another single-digit number appeared followed by another pair of squares. After each set of items, the child recalled the numbers by selecting the digits displayed on a 3x3 grid on the screen. Numbers 1–9 (except 7) were used. A number was not repeated within a list. List length ranged from 2 to 5 items with three trials at each list length. The outcome on this task was total digits recalled in correct order (maximum score was 42). The Cronbach’s *α* coefficient of internal consistency for this experimental task is .88 [61,62].

#### Attention control measures

*Flanker task*. This experimental task was developed as a measure of attention control. On each trial, children were presented with a cross in the center of the computer screen for 250 ms. This was followed by 5 arrows horizontally arranged in the center of the screen. Children were asked to focus on only the middle arrow and decide if the arrow pointed to the left or right by touching the word Left/Right on the screen as quickly as possible. On congruent trials the target arrow was flanked by arrows pointing in the same direction as the center arrow on each side. In the incongruent trials the middle arrow was flanked by the arrows pointing in the opposite direction as the middle arrow. Finally, on the neutral trials the arrow was flanked by a diamond shape made of two conjoined arrows on each side. All trial types were randomly presented 70 times each. The dependent variable was the reaction time difference between the incongruent and the congruent trials. The Cronbach’s *α* coefficient of internal consistency for this task was .97.

*Dichotic digits selective attention task*. The dichotic listening task was originally developed by [63] to measure selective attention. Children were presented with digit triplets simultaneously to both ears using a headphone. Digits one through nine (excluding seven because it was bisyllabic) spoken by a female speaker in standard American English were used. Identical digits did not occur at the same time in both ears. Digits were time-aligned such that they began and ended exactly at the same time in each ear. The intensity of the digits presented to the two ears was the same and it was 75 dB SPL. At the beginning of each trial, the child was prompted to pay attention to the randomly selected ear and ignore the digits presented to the opposite ear. The ear to-be-attended to was indicated by a beep in that ear simultaneous with an arrow on the screen pointing in that direction. Half of the trials were directed to right ear. Children recalled the digits by touching a 3x3 grid on the computer screen. On each trial, three-digit pairs were presented with an inter-stimulus interval of 500 ms and children were instructed to recall the target digits (i.e., from the ear to be attended) in the order presented. A total of 30 trials were used to measure dichotic selective attention ability in this task. The dependent measure for this task was the total number of digits recalled correctly in the same serial position as presented. The Cronbach’s *α* coefficient of internal consistency for this task was .94.

#### Vocabulary knowledge

*Receptive vocabulary*. The *Comprehensive Receptive and Expressive Vocabulary Test* (CREVT-3) [64] was used to measure children’s knowledge of the single words and their ability to use word associations. A spoken word was presented with a template of six pictures. The child was asked to point to the picture that best matched the spoken word. Each template represented a specific category such as play, occupations, animals, etc. There were ten categories with a variety of stimulus words for each. One point was given for every correct response. The stop rule within a category was two consecutive zero scores. The outcome score was total accuracy.

*Expressive vocabulary*. This test from the CREVT-3 [64] assessed children’s ability to describe the meaning of stimulus words. Each stimulus word was embedded in question form asking the child to describe the meaning of the target word. The score form listed a range of correct keywords and acceptable responses. If an incorrect or vague response was given, a standard prompt was provided to give the child a second chance to tell more about the target word. Each correct response, with or without the standard prompt, was given a score of one. The examiner discontinued the test when the child obtained three consecutive zero scores. The outcome score was total accuracy.

#### Retrieval from LTM

*Retrieval fluency*. This measure from the *Woodcock Johnson III Test of Cognitive Abilities* [65] is classified as a measure of retrieval from LTM or ideational fluency. Children were asked to name quickly as many exemplars as possible within a category such as animals, in one minute. Three categories were included (animals, first names, food/drink items). Accurately named exemplars were added to obtain the total score. Any repeated or incorrect exemplar received a score of 0.

*LTM retrieval—semantic priming task*. The ability to access available LTM was measured from category priming effects on a task where children were asked to retrieve items from semantic memory [66]. Standard instructions were given, and a practice trial was completed and repeated once if needed. First, children heard five monosyllabic words from two semantic categories (e.g., “*cat*, *bus*, *dog*, *truck*, *boat*”). Next, they answered which category items were more in number by a touch-screen response selection (“*Were there more animals or vehicles*?”). Last, they heard word pairs and judged whether both words belonged to the same semantic category or not. Words from the original five presented words, semantically related unprimed words, and unrelated unprimed words were used. Example word-pairs: Primed Direct: *boat-truck*, *dog-bus*; Primed-Indirect: *pig-cow*, *car-mouse*; Unprimed: *slide-swing*; *cap-pie*. Children made their category judgements by touching on the screen a box labeled SAME or a box labeled DIFFERENT. There were 8 primed (4 direct and 4 indirect) and 8 unprimed word pairs. There were three sets of five words and associated trials. Accuracy and response time for the semantic category judgments for the three conditions were obtained. Cronbach’s *α* coefficient of internal consistency for this LTM retrieval task is .80 for accuracy and .94 for response time data, respectively.

### Analytic approach

Aggregated percent correct recognition of words was subjected to a 2x2x2 factorial repeated measures analysis of variance (RM-ANOVA) to investigate a potential three-way interaction between word type (monosyllabic vs multisyllabic), lexical difficulty (easy vs. hard), and interruption rate (2.5 Hz vs. 5 Hz). Post-hoc contrasts utilized the Bonferroni correction for multiple comparisons. Paired t-test was performed to analyze the effect of rate (2.5 Hz vs. 5 Hz) on keyword recognition in sentences.

To investigate effects of the subject-specific continuous measures (working memory, attention, vocabulary, and LTM retrieval) on word recognition and keyword recognition in sentences, generalized “logistic” linear mixed effects models (GLMMs) were fit. Four subject-specific continuous measures were formed by averaging scores of multiple tasks; First, z scores of nonword repetition and digit working memory scores were averaged to form a composite measure of working memory capacity (WMC). Second, outcome measures from WJ-III retrieval fluency, accuracy of primed direct and primed indirect scores from LTM retrieval tasks were averaged to form a composite LTM retrieval score. Third, receptive and expressive vocabulary scores from CREVT-3 were combined to form a composite vocabulary measure. Finally, outcomes from the dichotic digits task and flanker task were combined to form a controlled attention measure.

Using GLMM is advantageous because participants with partial data can be incorporated. In addition, the hierarchical nature of the repeated measures is captured more accurately and correctly modeled with random effects thereby avoiding inflation of error rates and spurious results. A series of 2-level, random intercept nested models were fit based on the theoretical framework of the study and the likelihood ratio test was used to assess the significance of model terms [67]. Analysis was conducted in R 3.6.1 [68] and the `glmer()`function in the `lme4`package [69] was utilized for the GLMM analysis. A significance level of .05 was applied unless otherwise stated. S1 File include a documentation of the R code and the output.

## Results

One child had missing data (due to attrition) on some of the measures but could be included in the GLMM analysis. Another child had missing data on the working memory span task due to technical error. Summary statistics for subject specific measures are presented in Tables 1 and 2.

**Table 1:** Summary statistics for subjects-specific independent measures.

	*N*	M (SD)	Min	Max
Age, years	67	9.86 (1.25)	8.00	11.92
***Working Memory***				
NWR	66	88.11 (5.64)	60.00	96.00
Digit WM	65	39.42 (11.25)	9.00	59.00
***Long-term memory retrieval***				
WJ III Retrieval Fluency	66	54.71 (11.18)	37.00	84.00
LTM Primed Direct	66	11.03 (0.98)	8.00	12.00
LTM Primed Indirect	66	11.27 (1.14)	8.00	12.00
***Vocabulary***				
CREVT-Receptive	66	40.98 (8.27)	21.00	62.00
CREVT-Expressive	66	14.48 (3.89)	1.00	23.00
***Attention***				
Flanker	67	251.98 (174.99)	1.69	974.86
Dichotic Digit	67	67.76 (14.94)	29.00	88.00

***Note*:** NWR = Non-Word Repetition; WJ = Woodcock Johnson; LTM = Long-Term Memory; CREVT = Comprehensive Receptive and Expressive Vocabulary Test.

**Table 2:** Summary of percent of words correct and sentence keywords correct by interruption rate.

Stimulus Type		2.5 Hz	5 Hz
		M (SD)	M (SD)
Words: Lexically Easy	Mono-Syllabic	84.33 (1.04)	81.05 (1.00)
	Multi-Syllabic	95.37 (0.91)	86.72 (1.42)
Words: Lexically Hard	Mono-Syllabic	71.05 (1.26)	67.69 (1.30)
	Multi-Syllabic	74.48 (1.32)	81.34 (1.41)
Sentence: Keywords		78.25 (0.83)	91.32 (0.52)

As shown in Table 2, high frequency words with sparse lexical neighborhood density (i.e., lexically easy words) were better recognized than low frequency words with dense lexical neighborhood density (i.e., lexically hard). The effect of frequency of interruption was significantly different for recognition of multi- and monosyllabic words. Overall, lexically easy mono- and multisyllabic words were better recognized at 2.5 Hz than at 5 Hz whereas the reverse was noted for lexically hard multisyllabic words and recognition of keywords in sentences.

**Fig 2:**
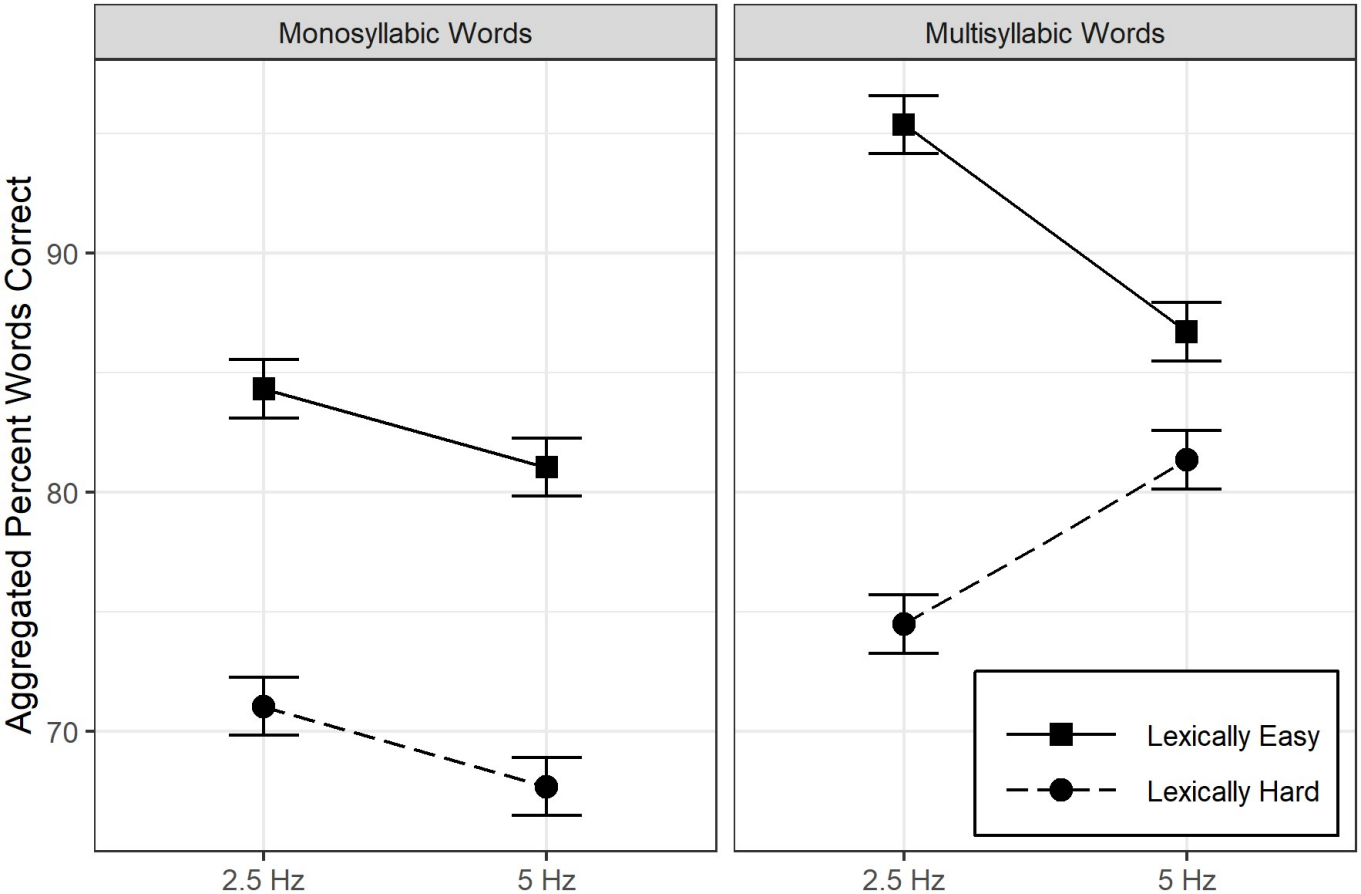
Illustration of the nature of interaction between word type (monosyllabic vs multisyllabic), lexical difficulty (easier vs. harder), and interruption rate (2.5 Hz vs. 5 Hz) on percent of words correct. Error bars represent +/- 1 SE.

RMANOVA revealed a three-way interaction between word type, lexical difficulty, and rate, *F*(1, 66) = 23.22, $\eta_{p}^{2}=.26$. The estimated marginal means are displayed in Fig 2. For monosyllabic words, no interaction was found between difficulty and rate, *t*(131) = 0.03, *p* > .999, rather participants were 26.64% (SE = 2.44) more accurate on easy words than hard words, *t*(130) = 10.91, *p* < .001, d = 2.67, but responded correctly 6.64% (SE = 2.10) lower at 5 Hz vs. 2.5 Hz, *t*(131) = 3.17, *p* = .011, d = 0.67. Conversely, among multisyllabic words, a two-way interaction was established, *t*(131) = 7.07, *p* < .001 such that even though participants did better on easy words at both rates, the gap {20.90% (SE = 1.64), *t*(258) = 12.73, *p* < .001, d = 2.09}, was larger at 2.5 Hz, than that (5.37% gap) at 5 Hz, *t*(258) = 3.27, *p* = .007, d = 0.54. Paired t-tests revealed, as expected, that sentence keyword recognition was significantly better at 5 Hz compared to 2.5 Hz, *t*(65) = 19.72, *p* < .001 (difference: M = 13.07, *95% CI* [11.75, 14.40], Cohen’s d = 2.43).

### GLMMs results for words

**Fig 3:**
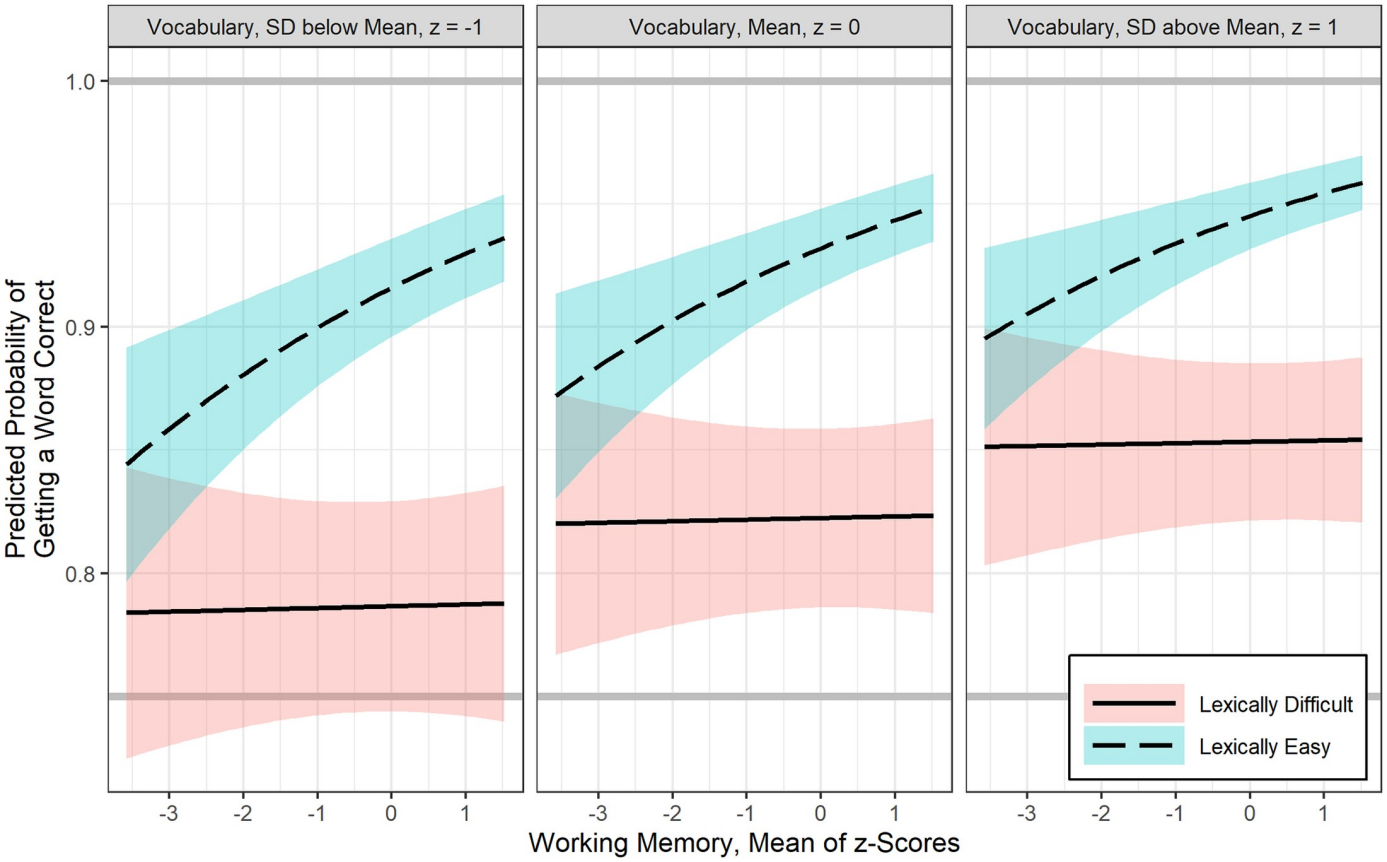
Estimated fit lines of a final (generalized) logistic linear mixed effects model (Model 1) for the probability of correct recognition of a lexically easy and difficult word, with 95% confidence bands. To illustrate the interaction between working memory capacity and lexical difficulty, mean and +/- 1SD breaks were chosen for vocabulary (standardized composite).

Parameter estimates for the best fitting GLMMs for the probability of word correct recognition is shown in Table 3. In logistic regression models the regression coefficient (b) is the estimated increase in the log odds of the outcome (i.e., correct identification of words in our models). The exponential function of regression coefficient, exp(b), is the odds ratio (OR) associated with one unit increase in the predictor variable. The model suggests that, controlling for vocabulary, there is a positive association between WMC and word recognition for the lexically easy words, b = 0.20, *p* < .05, however WMC significantly interacts with lexical difficulty of words, b = -0.19, *p* < .05. Fig 3 illustrates the interaction of lexical difficulty and the role of WMC. During recognition of lexically easy words, children with mean vocabulary (z = 0) and with the mean WMC (z = 0) have 13.7 odds or 93.2% chance of correctly recognizing any given lexically easy words (13.7/14.7 = .932). This also predicts that odds of recognizing any lexically hard words is 82.3%, OR = exp (2.62–1.08) = 4.66. This is shown as the height of the dashed line and solid line in the center panel of Fig 3 above the WMC value of zero (grand mean). Furthermore, after controlling for vocabulary, one standard deviation increase in WMC is associated with 1.22 odds of recognizing lexically easy words, OR = exp (0.20) = 1.22. This is evident by the consistency of the dashed lines increasing from left-to-right, for each of the three panels displaying illustrative vocabulary at mean and +/- 1 SD. However, the solid lines representing recognition of lexically hard words are relatively flat that shows that one standard deviation increase in WMC is associated with a nearly 0 odds of recognizing lexically hard words, OR = exp (-0.19 +0.20) = 0.01.

**Table 3:** Parameter estimates for best fit generalize “logistic” linear mixed effects models for word recognition.

	Model 1 (Best fit)		
**Fixed Effects**	b (SE)	OR	95% CI
Intercept	2.62 (0.25)***	13.70	[8.30, 22.61]
Main Effects			
Difficulty: Easy Vs. Hard	-1.08 (0.35)**	0.34	[0.17, 0.68]
WMC	0.20 (0.08)*	1.22	[1.04, 1.42]
Vocabulary	0.23 (0.06)***	1.26	[1.12, 1.41]
Cross-Level Interactions			
WMC X Difficulty	-0.19 (0.08)*	0.83	[0.70, 0.97]
**Random Effects**	Var		
Intercepts	0.07		
**Sample Size**	*N*		
Level 2 macro-units (Children)	66		
Level 1 micro-units (Words)	7920		

*** *p* < 0.001;

** *p* < 0.01;

* *p* < 0.05

**Fig 4:**
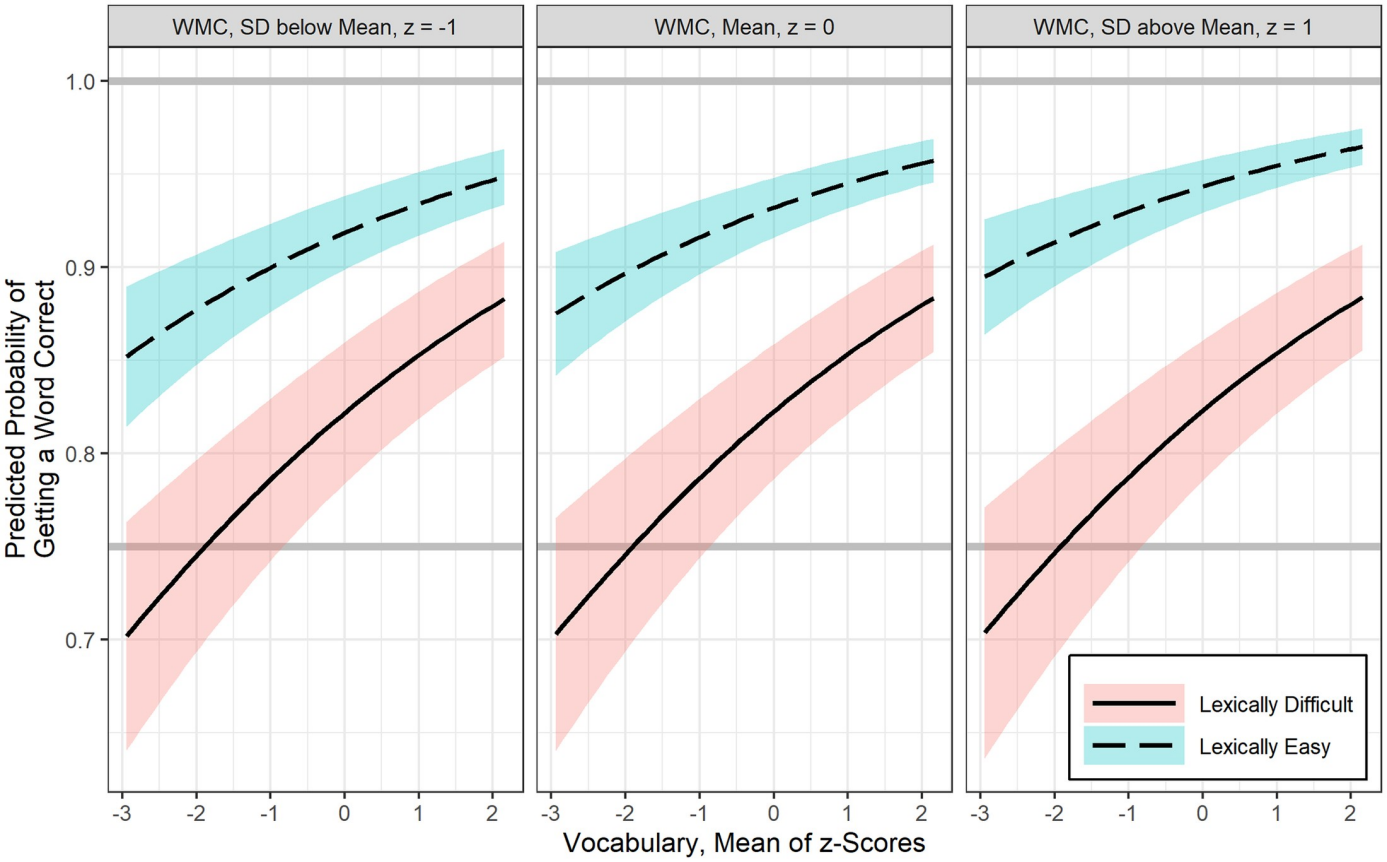
Estimated fit lines of a final (generalized) logistic linear mixed effects model (Model 1) for the probability of correct recognition of a lexically easy and difficult word, focusing on the effect of vocabulary at three levels of working memory capacity (mean and +/- 1SD), with 95% confidence bands.

After controlling for WMC, vocabulary was also positively related to recognition of lexically easy words, b = 0.23, *p* < .001. Fig 4 shows that irrespective of lexical difficulty, and after controlling for WMC, one SD increase in vocabulary is associated with 1.26 odds of increase in recognition of words. However, the interaction between lexical difficulty and vocabulary was not significant.

### GLMMs results for sentences

**Fig 5:**
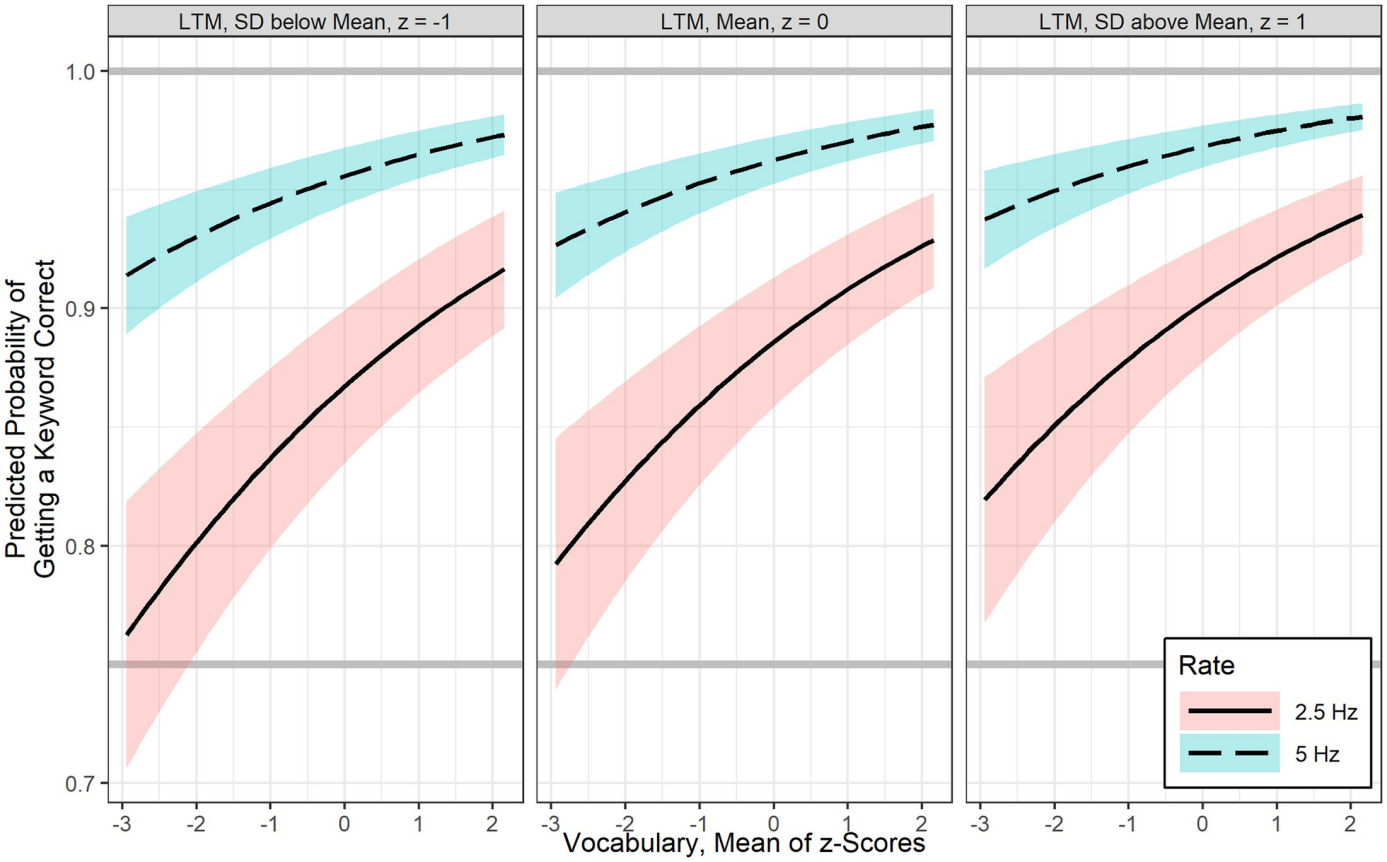
Estimated fit lines of a final (generalized) logistic linear mixed effects model (Model 2) for the probability of correct sentence keyword recognition at 2.5 Hz and 5 Hz interruption rate, focusing on the effect of vocabulary at three levels of long term memory retrieval ability (mean and +/- 1SD), with 95% confidence bands.

Best fitting GLMMs for the probability of keyword recognition in sentences is shown in Table 4. WMC was not significantly related to sentence keyword recognition. After controlling for LTM retrieval, there was a positive association between vocabulary and sentence keyword recognition at 2.5 Hz interruption rate, b = 0.24, *p* < .001, however vocabulary was not found to interact significantly with interruption rate. In general, as shown in Fig 5, one SD increase in vocabulary is associated with 1.27 odds of increase in sentence keyword recognition, regardless of interruption rate.

**Table 4:** Parameter estimates for best fit generalize “logistic” linear mixed effects models for sentence keyword recognition.

	Model 2 (Best fit)		
**Fixed Effects**	Co-efficient (SE)	OR	95% CI
Intercept	2.05 (0.27)***	7.76	[4.54, 13.24]
Main Effects			
Rate	1.20 (0.38)**	3.31	[1.56, 7.03]
LTM Retrieval	0.17 (0.07)*	1.19	[1.03, 1.38]
Vocabulary	0.24 (0.06)***	1.27	[1.13, 1.43]
**Random Effects**	Var		
Intercepts	0.11		
**Sample Size**	*N*		
Level 2 macro-units (Children)	66		
Level 1 micro-units (Keywords / Sentence)	16368		

*** p < 0.001;

** p < 0.01;

* p < 0.05

**Fig 6:**
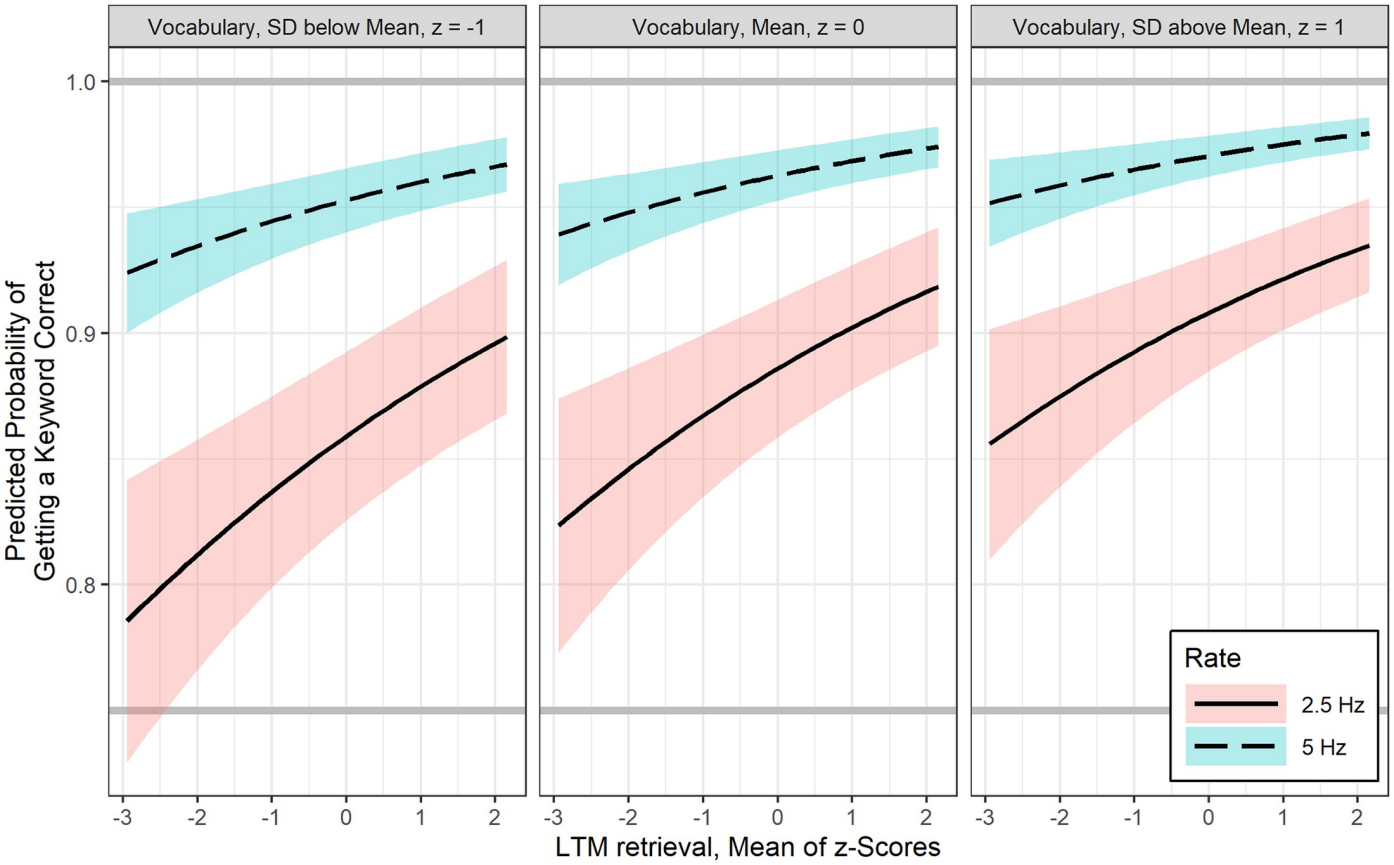
Estimated fit lines of a final (generalized) logistic linear mixed effects model (Model 2) for the probability of correct sentence keyword recognition at 2.5 Hz and 5 Hz interruption rate, focusing on the effect of long term memory retrieval ability at three levels of vocabulary (mean and +/- 1SD), with 95% confidence bands.

Similarly, after controlling for vocabulary, LTM retrieval was also positively related to sentence keyword recognition at 2.5 Hz, b = 0.17, *p* < .05. There was also no significant interaction between interruption rate and LTM retrieval ability. Fig 6 shows that one *SD* increase in LTM retrieval ability has 1.19 odds of increase in sentence keyword recognition.

## Discussion

The aim of this empirical research was to predict children’s auditory closure ability using cognitive and linguistic factors that are considered important for speech perception in adverse listening situations. Cognitive predictors were working memory and attention control ability. Linguistic predictors were lexical knowledge and LTM retrieval ability. Children’s auditory closure ability was measured using sentences and words interrupted at 2.5 Hz and 5 Hz interruption rates. At 2.5 Hz rate, children heard at least ***three*, *200 ms*** glimpses of clean speech segments per second to recognize words. Whereas at 5 Hz, ***five*, *100 ms*** glimpses of clean speech segments were available. The frequency and duration of clean glimpses determined by each interruption rate differentially influenced perceptual restoration of isolated words and keywords in sentences.

### Auditory closure for isolated words

As expected, high frequency words with sparse lexical neighborhood density (i.e., lexically easy words) were better recognized than low frequency words with dense lexical neighborhood density (i.e., lexically hard) [35,36,45]; Table 2, Fig 2). Overall, lexically easy mono- and multisyllabic words were better recognized at 2.5 Hz than at 5 Hz. This suggested that availability of more frequent glimpses (as determined by the higher interruption rate) did not contribute to better recognition of lexically easy words but rather led to more potential errors. There was no significant interaction between interruption rate and difficulty for monosyllabic words, however, lexical difficulty did interact with rate for multisyllabic words.

In a dense phonemic neighborhood, phonemic similarity between words leads to greater possibility of confusions and thereby erroneous word recognition. This is also a source of lexical competition because when words with similar phonemic onset in the child’s repertoire get activated, they inhibit each other towards recognition. For lexically easy words, within a low phonemic neighborhood density, greater glimpses did not serve to reduce lexical competition (likely because competing words were already few) and perhaps resulted in limited “spreading activation” of words. Limited spreading activation is expected to result in reduced overall lexical activation. In addition, because children’s inventory of lexically easy words is generally large (e.g., Tier 1 basic words), the need for lexical search may have been greater for lexically easy words when presented in isolation, thereby resulting in poorer performance with increased interruption rate. The same pattern of poorer performance with increased interruption rate was also observed for monosyllabic lexically hard words which had low word frequency and high phonemic neighborhood density. For monosyllabic words as they have a short overall duration, high neighborhood density can lead to greater lexical competition and thereby result in more confusions. Importantly, recognition of isolated open-set words not supported by any context is challenging and can be made worse by greater glimpses of partial information as suggested by the current data. Furthermore, 2.5 Hz may have been a better rate for recognition of monosyllabic words (in both lexically easy and hard conditions) than 5 Hz because at 2.5 Hz a larger chunk of the initial word segment was always available and initial gating of words generally results in superior performance [35]. However, at 5 Hz on monosyllabic words, any additional information potentially caused greater lexical confusion (Fig 2, Left Panel).

The advantage of increased glimpses was observed only for multisyllabic lexically hard words and this result was expected (Fig 2, Right Panel). That is, increased rate proved to be most facilitative when a word was multisyllabic and had high phonemic neighborhood density, the condition with the greatest phonemic and lexical demands. Therefore, more glimpses may have helped overcome lexical competition given the greater number of syllables. Interestingly, the influence of interruption rate on multisyllabic lexically hard words was similar to the effect of rate on sentences. This suggested a similar trend in the facilitative influence of greater number of glimpses on content that was increasing in complexity. The overall pattern of word recognition results obtained in children in this study are similar to that observed in adults [35].

### Auditory closure of words in sentences

Unlike words, sentences provided some linguistic context for recognition. Consequently, on sentences, increase in frequency of interruptions significantly improved speech recognition scores. Multiple frequent glimpses of clean speech at 5 Hz interruption led to better restoration of missing speech compared to 2.5 Hz. This result is well established and consistent with results from the adult and child literature [14,35,70].

### Factors influencing auditory closure ability

Consistent with our initial hypothesis that language ability rather than attention or WMC is crucial for restoring missing speech information, analyses suggested that auditory closure of missing speech in sentences, irrespective of the rate of interruption, was predicted by children’s lexical knowledge and their ability to accurately retrieve information from available LTM. Children with a larger lexicon performed significantly better in restoring words in sentences regardless of their working memory and attention control abilities. This specific finding is also consistent with results of Walker and colleagues [49] who studied time-gated speech recognition in children with mild to moderate hearing loss. Walker et al. [49] found that vocabulary, not children’s verbal WMC, mediated the relation between audibility and time-gated word recognition. An important aspect of the current study results, especially related to real-time spoken information processing is that, not only a larger lexicon helps, but also the ability to access that information in LTM is indeed crucial for filling-in missing speech information (Figs 5 and 6). Furthermore, the use of amplitude modulated speech shaped noise to fill the silent gaps may have provided bottom-up envelope cues contributing to children’s perceptual restoration ability. This inference is based on findings from adults where increased perceptual restoration was found when silent intervals were filled with envelope modulated noise relative to stochastic noise [59,71]. Bottom-up temporal integration of envelope cues along with integrating glimpses of clean speech are useful to auditory closure ability.

However, the GLMM prediction models were not as expected for isolated word recognition (i.e, LTM retrieval did not predict word recognition). It is well known that spoken word recognition is influenced by multiple lexical factors such word frequency and lexical neighborhood density. Accordingly, lexical knowledge was a strong predictor of auditory closure of words at both interruption rates. Working memory was also associated with auditory closure of only lexically easy words (see Fig 3). LTM retrieval ability and attention did not correlate with auditory closure of words. It is possible that children show greater lexical effects on word recognition in degraded listening conditions especially when the noise is relatively higher than target speech [45]. Furthermore, recognition of isolated words even without any context does appear to be advantaged by cognitive and linguistic resources.

These results highlight the importance of strong lexical networks to maximize speech understanding in adverse listening situations. Children with a larger lexicon recovered missing speech much better than children with low vocabulary scores (see Figs 4 and 5). These results can be linked to speech perception in noise studies in normal hearing children [45], children with hearing loss [36,49], dyslexia [5,34], and specific language impairment [72]. Relative to typically developing children, children with weak language systems do exhibit significant difficulty in understanding speech in adverse listening situations. Accordingly, a stronger relationship between language ability and listening has been observed in clinical populations when compared to typically developing children [61,73]. Significant difficulty understanding speech in noise is commonly reported in children suspected to have APD [66]. Results of the current study indicate an area of assessment and intervention that may potentially benefit a larger group of children with listening difficulties, not just children with hearing loss. That is, findings from the current study and from several related studies in children [34,49] suggest that intervention targeted to strengthen lexical networks and access to LTM can potentially help children combat the deleterious effect of pervasive noise in their learning environments. Given that listening in complex auditory environments is common in everyday life, restoration of missing speech is a crucial ability that needs to be facilitated in children.

## Conclusions

We examined auditory closure ability in children using lexically easy words, lexically hard words and sentences at two interruption rates (2.5 Hz and 5 Hz) in noise-filled condition. Influential factors included in the modeling were vocabulary knowledge, retrieval from LTM, attention, and working memory. Novel findings were related to the significant interaction between rate of interruption, neighborhood density of words, and type of words. Lexical difficulty significantly interacted with auditory closure of multisyllabic words, but not monosyllabic words. Lexically easy mono- and multisyllabic words were better recognized at the lower interruption rate (2.5 Hz) than at the higher rate (5 Hz). Greater number of glimpses were advantageous only for recognition of multisyllabic lexically hard words and sentences. For both words and sentences, lexical knowledge was significantly associated with auditory closure ability. Furthermore, the ability to retrieve information from LTM (not attention/WMC) was crucial for recognition of keywords in sentences. Working memory was only associated with recognition of lexically easy words. LTM retrieval ability and attention were not associated with auditory closure of words. Overall, findings indicated the importance of vocabulary knowledge and LTM retrieval ability in maximizing speech recognition in adverse listening situations. Therefore, lexical knowledge and LTM retrieval ability are critical constructs of relevance for children who are reported to have listening difficulties.

## Supporting information

S1 File (HTML)
