# Supplementary material for: Auditory processing in children: Role of working memory and lexical ability in auditory closure
Source: PLoS One. 2020 Nov 4;15(11):e0240534. doi: 10.1371/journal.pone.0240534 (PMC7641369; doi:10.1371/journal.pone.0240534)
Supplement: S1 File — (HTML) [file pone.0240534.s001.html]

Auditory Processing in Children: Role of Working Memory and Lexical Ability in Auditory Closure


# Auditory Processing in Children: Role of Working Memory and Lexical Ability in Auditory Closure

### Statistical Analysis

#### Dr. Naveen K. Nagaraj & Dr. Beula M. Magimairaj, Cognitive Hearing Science Lab, email: naveen@usu.edu

#### October 02, 2020

- Packages
  - CRAN
  - GitHub
- Data Analysis for Words
  - Import Data
    - Participant-Level Data
    - Experimental Data Words
    - Merge Together
    - Aggregate Data
- Exploratory Data Analysis
  - Summary Statistics
    - Indepdent Variables
  - Summary of Independent Variables
    - Aggregated Outcome: Percent of Words Correct
  - Bivariate Correlations Between Measures
    - Working Memory
    - Long Term Memory
    - Vocabulary
    - Attention
    - All Independant Variables for Modeling
  - Visualize Aggregated Data
    - Means Plot (Aggregated DV)
- 2 x 2 x 2 RM ANOVA on Aggregated Data
  - Fit Model
  - Omnibus F-tests, Type III SS
  - Interaction Plot, Estimated Marginal Means
  - Post Hoc t-Tests
    - Pooled Standard Deviation
    - Estimated Marginal Means
    - Contrasts: difficulty X rate, within type of word
- GLMM: Generalized (Logistic) Linear Mixed Effects Regression
  - Null Model
    - Variance Components
    - ICC: intraclass-correlation
  - Investigate 3-way Interaction: Rate x Type x Difficulty
    - Fit Nested Models
    - Likelihood Ratio Tests: Compare Nested Models
    - Table of Parameter Estimates
  - Final Model
    - Table of Parameter Estimates
    - Interaction Plot
- Data Analysis for Sentences
  - Import Data
    - Experimental Data
    - Merge together
    - Aggregate Data
- Exploratory Data Analysis
  - Summary Statistics: Aggregated Outcome: Percent Correct by Interruption Rate
  - Paired t-test Effect size
  - Visualization of Aggregated Data
- GLMM = Generalized (Logistic) Linear Mixed Effects Regression
  - ICC: intraclass-correlation
  - Final Model: Parameter Estimates
  - Model Plots

# Packages

## CRAN

Data Tools

```
library(magrittr)     # forward pipe operators for R
library(tidyverse)    # Loads several core packages
library(readxl)       # Importing Excel
```

Basics Tools

```
library(GGally)       # Extension to 'ggplot2' (ggpairs)
library(corrplot)     # Visualize correlation matrix
library(psych)        # Handy tid-bits
library(furniture)    # Tables, descriptive
library(texreg)       # Tables, models
```

Multilevel Modeling Tools

```
library(lme4)         # Linear, generalized linear, & nonlinear mixed models
library(lmerTest)     # Tests on lmer objects
library(HLMdiag)      # Diagnostic Tools for for nlme & lmer4
library(sjstats)      # ICC calculations
library(optimx)       # Different optimizers to solve mlm's
library(rsq)          # R-squared and related measures
library(effectsize)   # effect size calculations
```

Structural Equation Modeling (including CFA)

```
library(lavaan)
library(lavaanPlot)
```

## GitHub

```
#library(devtools)
#devtools::install_github("SarBearSchwartz/texreghelpr")

library(texreghelpr)  # Exponentiate logistic models for texreg tables
```

# Data Analysis for Words

## Import Data

### Participant-Level Data

Sample Size: participants, N = 67

```
data_sub <- readxl::read_excel("ISP_Master_ISPpaper.xlsx") %>% 
  dplyr::rename_all(tolower) %>% 
  dplyr::mutate_all(as.numeric) %>% 
  dplyr::mutate(ageyears = agemonths/12) %>% 
  dplyr::mutate(id = factor(id)) %>% 
  tidyr::pivot_longer(cols= c(nwr, 
                              digit_wm_sum,                   
                              wj3_rf_raw,
                              flanker_rt,
                              dd_quiet,
                              ltm_acc_sum_primed_direct,
                              ltm_acc_sum_primed_indirect, 
                              crevtr_raw, 
                              crevte_raw),
                      names_to = "measure",
                      values_to = "orig") %>% 
  dplyr::group_by(measure) %>% 
  dplyr::mutate(z = scale(orig)) %>% 
  dplyr::ungroup() %>% 
  tidyr::pivot_wider(names_from = measure,
                     values_from = c(orig, z),
                     names_sep = "_") %>% 
  dplyr::mutate(zmean_wm    = furniture::rowmeans(z_nwr, 
                                                  z_digit_wm_sum, 
                                                  na.rm = TRUE),
                zmean_ltm   = furniture::rowmeans(z_ltm_acc_sum_primed_indirect, 
                                                  z_ltm_acc_sum_primed_direct, 
                                                  z_wj3_rf_raw, 
                                                  na.rm = TRUE),
                zmean_vocab = furniture::rowmeans(z_crevtr_raw,
                                                  z_crevte_raw, 
                                                  na.rm = TRUE),
                zmean_attn  = furniture::rowmeans(z_dd_quiet, 
                                                  -z_flanker_rt,                 
                                                  na.rm = TRUE)) 

tibble::glimpse(data_sub)
```

```
Rows: 67
Columns: 27
$ id                               <fct> 300, 301, 302, 303, 304, 305, 306,...
$ agemonths                        <dbl> 127, 121, 141, 101, 128, 121, 102,...
$ wj3_ws_raw                       <dbl> 12, 19, 17, 16, 13, 16, 14, 18, 17...
$ flanker_prop                     <dbl> 1.20, 1.02, 1.10, 1.19, 1.05, 1.43...
$ ageyears                         <dbl> 10.583333, 10.083333, 11.750000, 8...
$ orig_nwr                         <dbl> 60, 91, 86, 88, 86, 90, 90, 92, 93...
$ orig_digit_wm_sum                <dbl> 15, 49, 37, 28, 40, 39, 32, 53, 47...
$ orig_wj3_rf_raw                  <dbl> 42, 53, 57, 41, 71, 70, 53, 49, 57...
$ orig_flanker_rt                  <dbl> 214.04, 26.07, 81.86, 203.65, 49.6...
$ orig_dd_quiet                    <dbl> 34, 87, 84, 73, 70, 70, 69, 65, 83...
$ orig_ltm_acc_sum_primed_direct   <dbl> 9, 12, 10, 9, 11, 12, 11, 12, 10, ...
$ orig_ltm_acc_sum_primed_indirect <dbl> 10, 12, 11, 9, 12, 12, 12, 12, 12,...
$ orig_crevtr_raw                  <dbl> 41, 50, 48, 37, 48, 41, 47, 52, 49...
$ orig_crevte_raw                  <dbl> 10, 16, 13, 11, 12, 17, 15, 14, 16...
$ z_nwr                            <dbl[,1]> <matrix[26 x 1]>
$ z_digit_wm_sum                   <dbl[,1]> <matrix[26 x 1]>
$ z_wj3_rf_raw                     <dbl[,1]> <matrix[26 x 1]>
$ z_flanker_rt                     <dbl[,1]> <matrix[26 x 1]>
$ z_dd_quiet                       <dbl[,1]> <matrix[26 x 1]>
$ z_ltm_acc_sum_primed_direct      <dbl[,1]> <matrix[26 x 1]>
$ z_ltm_acc_sum_primed_indirect    <dbl[,1]> <matrix[26 x 1]>
$ z_crevtr_raw                     <dbl[,1]> <matrix[26 x 1]>
$ z_crevte_raw                     <dbl[,1]> <matrix[26 x 1]>
$ zmean_wm                         <dbl> -3.57777241, 0.68268090, -0.294134...
$ zmean_ltm                        <dbl> -1.44325418, 0.49193346, -0.363029...
$ zmean_vocab                      <dbl> -0.575865456, 0.740066804, 0.23329...
$ zmean_attn                       <dbl> -1.02115165, 1.28917922, 1.0293961...
```

### Experimental Data Words

Sample Size: words, n = 67 participants x 120 words each = 8040 words

```
data_exp <- readxl::read_excel("ISP_Words_data.xlsx",
                               range = "A1:F8041") %>% 
  dplyr::rename_all(tolower) %>% 
  dplyr::mutate_at(vars(id,words), 
                   factor) %>% 
  dplyr::mutate(rate = rate %>% 
                  factor(levels = c("2.5", "5"),
                         labels = c("2.5 Hz", "5 Hz"))) %>% 
  dplyr::mutate(type = type %>% 
                  factor(levels = c("mono", "multi"),
                         labels = c("Monosyllabic Words",
                                    "Multisyllabic Words"))) %>% 
  dplyr::mutate(difficulty = difficulty %>% 
                  factor(levels = c("easy", "hard"),
                         labels = c("Lexically Easy",
                                    "Lexically Difficult")))

tibble::glimpse(data_exp)
```

```
Rows: 8,040
Columns: 6
$ id         <fct> 300, 300, 300, 300, 300, 300, 300, 300, 300, 300, 300, 3...
$ type       <fct> Monosyllabic Words, Monosyllabic Words, Monosyllabic Wor...
$ difficulty <fct> Lexically Easy, Lexically Easy, Lexically Easy, Lexicall...
$ rate       <fct> 2.5 Hz, 2.5 Hz, 2.5 Hz, 2.5 Hz, 2.5 Hz, 2.5 Hz, 2.5 Hz, ...
$ words      <fct> 2.5mone1, 2.5mone2, 2.5mone3, 2.5mone4, 2.5mone5, 2.5mon...
$ w_score    <dbl> 1, 1, 1, 1, 1, 1, 1, 1, 1, 1, 1, 1, 1, 1, 1, 1, 0, 1, 1,...
```

### Merge Together

```
data_long <- data_sub %>% 
  dplyr::right_join(data_exp, by = "id")

tibble::glimpse(data_long)
```

```
Rows: 8,040
Columns: 32
$ id                               <fct> 300, 300, 300, 300, 300, 300, 300,...
$ agemonths                        <dbl> 127, 127, 127, 127, 127, 127, 127,...
$ wj3_ws_raw                       <dbl> 12, 12, 12, 12, 12, 12, 12, 12, 12...
$ flanker_prop                     <dbl> 1.2, 1.2, 1.2, 1.2, 1.2, 1.2, 1.2,...
$ ageyears                         <dbl> 10.58333, 10.58333, 10.58333, 10.5...
$ orig_nwr                         <dbl> 60, 60, 60, 60, 60, 60, 60, 60, 60...
$ orig_digit_wm_sum                <dbl> 15, 15, 15, 15, 15, 15, 15, 15, 15...
$ orig_wj3_rf_raw                  <dbl> 42, 42, 42, 42, 42, 42, 42, 42, 42...
$ orig_flanker_rt                  <dbl> 214.04, 214.04, 214.04, 214.04, 21...
$ orig_dd_quiet                    <dbl> 34, 34, 34, 34, 34, 34, 34, 34, 34...
$ orig_ltm_acc_sum_primed_direct   <dbl> 9, 9, 9, 9, 9, 9, 9, 9, 9, 9, 9, 9...
$ orig_ltm_acc_sum_primed_indirect <dbl> 10, 10, 10, 10, 10, 10, 10, 10, 10...
$ orig_crevtr_raw                  <dbl> 41, 41, 41, 41, 41, 41, 41, 41, 41...
$ orig_crevte_raw                  <dbl> 10, 10, 10, 10, 10, 10, 10, 10, 10...
$ z_nwr                            <dbl[,1]> <matrix[26 x 1]>
$ z_digit_wm_sum                   <dbl[,1]> <matrix[26 x 1]>
$ z_wj3_rf_raw                     <dbl[,1]> <matrix[26 x 1]>
$ z_flanker_rt                     <dbl[,1]> <matrix[26 x 1]>
$ z_dd_quiet                       <dbl[,1]> <matrix[26 x 1]>
$ z_ltm_acc_sum_primed_direct      <dbl[,1]> <matrix[26 x 1]>
$ z_ltm_acc_sum_primed_indirect    <dbl[,1]> <matrix[26 x 1]>
$ z_crevtr_raw                     <dbl[,1]> <matrix[26 x 1]>
$ z_crevte_raw                     <dbl[,1]> <matrix[26 x 1]>
$ zmean_wm                         <dbl> -3.577772, -3.577772, -3.577772, -...
$ zmean_ltm                        <dbl> -1.443254, -1.443254, -1.443254, -...
$ zmean_vocab                      <dbl> -0.5758655, -0.5758655, -0.5758655...
$ zmean_attn                       <dbl> -1.021152, -1.021152, -1.021152, -...
$ type                             <fct> Monosyllabic Words, Monosyllabic W...
$ difficulty                       <fct> Lexically Easy, Lexically Easy, Le...
$ rate                             <fct> 2.5 Hz, 2.5 Hz, 2.5 Hz, 2.5 Hz, 2....
$ words                            <fct> 2.5mone1, 2.5mone2, 2.5mone3, 2.5m...
$ w_score                          <dbl> 1, 1, 1, 1, 1, 1, 1, 1, 1, 1, 1, 1...
```

### Aggregate Data

Sample Size: words, n = 67 participants x 8 (2 word types x 2 difficulties, x 2 rates) = 536 words

```
data_agg <- data_long %>% 
  dplyr::group_by(id, 
                  agemonths, ageyears,
                  zmean_wm,
                  zmean_ltm,
                  zmean_vocab,
                  zmean_attn,
                  rate, type, difficulty) %>% 
  dplyr::summarise(set_n = n(),
                   prop_correct = sum(w_score)/n()) %>% 
  dplyr::mutate(per_correct = 100 * prop_correct) %>% 
  dplyr::ungroup() %>% 
  dplyr::mutate(set = interaction(rate, type, difficulty) %>% 
                  as.numeric()) %>% 
  dplyr::arrange(id, set) %>% 
  dplyr::select(id, set, set_n, everything())


tibble::glimpse(data_agg)
```

```
Rows: 536
Columns: 14
$ id           <fct> 300, 300, 300, 300, 300, 300, 300, 300, 301, 301, 301,...
$ set          <dbl> 1, 2, 3, 4, 5, 6, 7, 8, 1, 2, 3, 4, 5, 6, 7, 8, 1, 2, ...
$ set_n        <int> 20, 20, 10, 10, 20, 20, 10, 10, 20, 20, 10, 10, 20, 20...
$ agemonths    <dbl> 127, 127, 127, 127, 127, 127, 127, 127, 121, 121, 121,...
$ ageyears     <dbl> 10.583333, 10.583333, 10.583333, 10.583333, 10.583333,...
$ zmean_wm     <dbl> -3.5777724, -3.5777724, -3.5777724, -3.5777724, -3.577...
$ zmean_ltm    <dbl> -1.4432542, -1.4432542, -1.4432542, -1.4432542, -1.443...
$ zmean_vocab  <dbl> -0.5758655, -0.5758655, -0.5758655, -0.5758655, -0.575...
$ zmean_attn   <dbl> -1.021152, -1.021152, -1.021152, -1.021152, -1.021152,...
$ rate         <fct> 2.5 Hz, 5 Hz, 2.5 Hz, 5 Hz, 2.5 Hz, 5 Hz, 2.5 Hz, 5 Hz...
$ type         <fct> Monosyllabic Words, Monosyllabic Words, Multisyllabic ...
$ difficulty   <fct> Lexically Easy, Lexically Easy, Lexically Easy, Lexica...
$ prop_correct <dbl> 0.95, 0.75, 1.00, 0.80, 0.50, 0.70, 0.90, 0.70, 1.00, ...
$ per_correct  <dbl> 95, 75, 100, 80, 50, 70, 90, 70, 100, 85, 90, 100, 85,...
```

# Exploratory Data Analysis

## Summary Statistics

### Indepdent Variables

```
data_sub %>% 
  dplyr::select(agemonths, ageyears, 
                starts_with("orig_"),
                starts_with("zmean")) %>% 
  psych::describe(skew = FALSE)
```

```
data_sub %>% 
  dplyr::select(agemonths, ageyears, 
                starts_with("orig_"),
                starts_with("zmean")) %>% 
  furniture::table1(digits = 3,
                    caption = "Summary of Independent Variables")
```

## Summary of Independent Variables

```
                              Mean/Count (SD/%)
                              n = 65
```

agemonths  
118.554 (15.163) ageyears  
9.879 (1.264)  
orig\_nwr  
88.108 (5.682)  
orig\_digit\_wm\_sum  
39.415 (11.248)  
orig\_wj3\_rf\_raw  
54.954 (11.087)  
orig\_flanker\_rt  
255.651 (175.857) orig\_dd\_quiet  
68.138 (14.266)  
orig\_ltm\_acc\_sum\_primed\_direct  
11.015 (0.976)  
orig\_ltm\_acc\_sum\_primed\_indirect  
11.262 (1.149)  
orig\_crevtr\_raw  
41.031 (8.323)  
orig\_crevte\_raw  
14.554 (3.877)  
zmean\_wm  
0.000 (0.841)  
zmean\_ltm  
-0.001 (0.755)  
zmean\_vocab  
0.012 (0.936)  
zmean\_attn  
0.002 (0.768)  
—————————————————-

```
data_sub %>% 
  dplyr::select(starts_with("orig_")) %>% 
  #summary()
describe()
```

```
data_sub %>% 
  dplyr::select(starts_with("zmean_")) %>% 
  summary()
```

```
    zmean_wm           zmean_ltm        zmean_vocab         zmean_attn     
 Min.   :-3.577772   Min.   :-2.1357   Min.   :-2.94286   Min.   :-2.1579  
 1st Qu.:-0.448834   1st Qu.:-0.3976   1st Qu.:-0.55692   1st Qu.:-0.5157  
 Median : 0.105043   Median : 0.1390   Median : 0.04076   Median : 0.1142  
 Mean   :-0.000143   Mean   : 0.0000   Mean   : 0.00000   Mean   : 0.0000  
 3rd Qu.: 0.549713   3rd Qu.: 0.5143   3rd Qu.: 0.48878   3rd Qu.: 0.5325  
 Max.   : 1.570595   Max.   : 1.4166   Max.   : 2.24508   Max.   : 1.2892  
 NA's   :1           NA's   :1         NA's   :1
```

```
zmean_vocab_qs <- data_sub %>% 
  dplyr::pull(zmean_vocab) %>% 
  quantile(probs = c(.25, .50, .75), 
           na.rm = TRUE) %>% 
  round(2)

zmean_vocab_qs
```

```
  25%   50%   75% 
-0.56  0.04  0.49
```

```
zmean_wm_qs <- data_sub %>% 
  dplyr::pull(zmean_wm) %>% 
  quantile(probs = c(.25,  .50, .75), 
           na.rm = TRUE) %>% 
  round(2)

zmean_wm_qs
```

```
  25%   50%   75% 
-0.45  0.11  0.55
```

```
zmean_ltm_qs <- data_sub %>% 
  dplyr::pull(zmean_ltm) %>% 
  quantile(probs = c(.25,  .50, .75), 
           na.rm = TRUE) %>% 
  round(2)

zmean_ltm_qs
```

```
  25%   50%   75% 
-0.40  0.14  0.51
```

### Aggregated Outcome: Percent of Words Correct

```
data_agg %>% 
  dplyr::group_by(difficulty, rate, type) %>% 
  dplyr::summarise(mean = mean(per_correct),
                   se   = sd(per_correct)/sqrt(n())) %>% 
  dplyr::mutate(cell = paste0(mean %>% round(3) %>% format(nsmall = 3), 
                              "  (", 
                              se %>% round(3) %>% format(nsmall = 3), 
                                     ")")) %>% 
  dplyr::select(-mean, -se) %>% 
  tidyr::pivot_wider(names_from = type,
                     values_from = cell) %>% 
  pander::pander(caption = "Percent of Words Correct, by Difficulty, Rate, and Type of Word")
```

Percent of Words Correct, by Difficulty, Rate, and Type of Word


| difficulty | rate | Monosyllabic Words | Multisyllabic Words |
| --- | --- | --- | --- |
| Lexically Easy | 2.5 Hz | 84.328 (1.036) | 95.373 (0.910) |
| Lexically Easy | 5 Hz | 81.045 (1.001) | 86.716 (1.417) |
| Lexically Difficult | 2.5 Hz | 71.045 (1.256) | 74.478 (1.316) |
| Lexically Difficult | 5 Hz | 67.687 (1.304) | 81.343 (1.409) |

## Bivariate Correlations Between Measures

### Working Memory

```
data_sub %>% 
  dplyr::select(zmean_wm,
                orig_nwr,
                orig_digit_wm_sum) %>% 
  furniture::tableC(na.rm = TRUE)
```

```
--------------------------------------------------------
                      [1]           [2]           [3]  
 [1]zmean_wm          1.00                             
 [2]orig_nwr          0.839 (<.001) 1.00               
 [3]orig_digit_wm_sum 0.837 (<.001) 0.405 (<.001) 1.00 
--------------------------------------------------------
```

### Long Term Memory

```
data_sub %>% 
  dplyr::select(zmean_ltm,
                orig_ltm_acc_sum_primed_indirect,
                orig_ltm_acc_sum_primed_direct,
                orig_wj3_rf_raw) %>% 
  furniture::tableC(na.rm = TRUE)
```

```
-------------------------------------------------------------------------------------
                                     [1]           [2]           [3]          
 [1]zmean_ltm                        1.00                                     
 [2]orig_ltm_acc_sum_primed_indirect 0.742 (<.001) 1.00                       
 [3]orig_ltm_acc_sum_primed_direct   0.791 (<.001) 0.419 (<.001) 1.00         
 [4]orig_wj3_rf_raw                  0.714 (<.001) 0.248 (0.045) 0.358 (0.003)
 [4]  
      
      
      
 1.00 
-------------------------------------------------------------------------------------
```

### Vocabulary

```
data_sub %>% 
  dplyr::select(zmean_vocab,
                orig_crevtr_raw,
                orig_crevte_raw) %>% 
  furniture::tableC(na.rm = TRUE)
```

```
------------------------------------------------------
                    [1]           [2]           [3]  
 [1]zmean_vocab     1.00                             
 [2]orig_crevtr_raw 0.934 (<.001) 1.00               
 [3]orig_crevte_raw 0.934 (<.001) 0.744 (<.001) 1.00 
------------------------------------------------------
```

### Attention

```
data_sub %>% 
  dplyr::select(zmean_attn,
                orig_dd_quiet,
                orig_flanker_rt) %>% 
  furniture::tableC(na.rm = TRUE)
```

```
--------------------------------------------------------
                    [1]            [2]            [3]  
 [1]zmean_attn      1.00                               
 [2]orig_dd_quiet   0.781 (<.001)  1.00                
 [3]orig_flanker_rt -0.781 (<.001) -0.219 (0.075) 1.00 
--------------------------------------------------------
```

### All Independant Variables for Modeling

```
data_sub %>% 
  dplyr::select(ageyears,
                starts_with("zmean_")) %>% 
  furniture::tableC(na.rm = TRUE)
```

```
------------------------------------------------------------------------------
                [1]           [2]           [3]           [4]           [5]  
 [1]ageyears    1.00                                                         
 [2]zmean_wm    0.161 (0.197) 1.00                                           
 [3]zmean_ltm   0.28 (0.023)  0.454 (<.001) 1.00                             
 [4]zmean_vocab 0.634 (<.001) 0.424 (<.001) 0.485 (<.001) 1.00               
 [5]zmean_attn  0.174 (0.162) 0.441 (<.001) 0.362 (0.003) 0.365 (0.003) 1.00 
------------------------------------------------------------------------------
```

```
pmat <- data_sub %>% 
  dplyr::select(ageyears,
                starts_with("zmean_")) %>% 
  cor.mtest() %$%
  p
```

```
data_sub %>% 
  dplyr::select("Age" = ageyears,
                "Working\nMemory" = zmean_wm,
                "LTM\nretrieval" = zmean_ltm,
                "Vocabulary" = zmean_vocab,
                "Attention" = zmean_attn) %>%
  cor(use = "pairwise") %>% 
  corrplot::corrplot.mixed(tl.col = "black",
                           cl.lim = c(0, 1),
                           upper.col = colorRampPalette(c("white", 
                                                          "white", 
                                                          "black"))(100),
                           lower.col = "black",
                           p.mat = pmat, 
                           sig.level = 0.05)
```

Correlation plot between age, WMC, attention, Vocabulary and LTM retrieval

## Visualize Aggregated Data

### Means Plot (Aggregated DV)

```
data_agg %>% 
  ggplot(aes(x = rate,
             y = per_correct,
             group = interaction(difficulty, rate))) +
  geom_violin(aes(fill = difficulty),
              alpha = .6,
              position = position_dodge(width = .6)) +
  stat_summary(fun.y = mean,
               geom = "point",
               position = position_dodge(width = .6))+
  stat_summary(aes(linetype = difficulty,
                   group = difficulty),
               fun.y = mean,    
               geom = "line",
               position = position_dodge(width = .6)) +
  theme_bw() +
  theme(legend.background = element_rect(color = "black"),
        legend.key.width = unit(1.75, "cm"),
        legend.position = c(1, 0),
        legend.justification = c(1.05, -0.1)) +
  labs(x = NULL,
       y = "Percentage of Words Correct",
       shape = NULL,
       fill = NULL,
       linetype = NULL) +
  scale_linetype_manual(values = c("solid", "longdash")) +
  scale_fill_manual(values = c("gray40", "gray80")) +
  scale_shape_manual(values = c(15, 19)) +
  facet_grid(~ type)  +
  coord_cartesian(ylim = c(30, 100))
```

Mean plots for word reconition by rate aand lexical difficulty

# 2 x 2 x 2 RM ANOVA on Aggregated Data

3-way Factorial Repeated Measures Analysis of Variance

## Fit Model

Repeated Measures:

- Rate: 2.5 vs. 5 Hz
- Type: mono vs. multi syllabolic
- Difficulty: Less vs. More

```
fit_rmaov_3way <- data_agg %>% 
  afex::aov_4(per_correct ~ 1 + (rate*type*difficulty|id),
              data = .,
              anova_table = list(es = c("ges", "pes")))
```

```
summary(fit_rmaov_3way)
```

```
Univariate Type III Repeated-Measures ANOVA Assuming Sphericity

                      Sum Sq num Df Error SS den Df    F value    Pr(>F)    
(Intercept)          3452034      1  13706.6     66 16622.2010 < 2.2e-16 ***
rate                     596      1   5220.1     66     7.5301  0.007807 ** 
type                    9571      1   5394.3     66   117.1061 2.883e-16 ***
difficulty             23446      1   7407.2     66   208.9097 < 2.2e-16 ***
rate:type                197      1   4493.6     66     2.8944  0.093596 .  
rate:difficulty         1999      1   4854.6     66    27.1712 2.006e-06 ***
type:difficulty            1      1   5777.0     66     0.0133  0.908463    
rate:type:difficulty    2037      1   5790.8     66    23.2207 8.827e-06 ***
---
Signif. codes:  0 '***' 0.001 '**' 0.01 '*' 0.05 '.' 0.1 ' ' 1
```

## Omnibus F-tests, Type III SS

Effect Sizes \* `ges` Generalized Eta-squared \* `pes` Partial Eta-squared

```
afex::nice(fit_rmaov_3way)
```

## Interaction Plot, Estimated Marginal Means

3-way interaction between: rate, type, and difficulty

```
fit_rmaov_3way %>% 
  emmeans::emmeans(~ rate*type*difficulty) %>% 
  data.frame() %>% 
  dplyr::mutate(rate = rate %>% 
                  forcats::fct_recode("2.5 Hz" = "X2.5.Hz", 
                                      "5 Hz" = "X5.Hz")) %>% 
  dplyr::mutate(type = type %>% 
                  forcats::fct_recode("Monosyllabic Words" = "Monosyllabic.Words",
                                      "Multisyllabic Words" = "Multisyllabic.Words")) %>% 
  dplyr::mutate(difficulty = difficulty %>% 
                  forcats::fct_recode("Lexically Easy"= "Lexically.Easy",
                                      "Lexically Hard" = "Lexically.Difficult")) %>% 
  ggplot(aes(x = rate,
             y = emmean,
             shape = difficulty)) +
  geom_errorbar(aes(ymin = emmean - SE,
                    ymax = emmean + SE),
                width = .25) +
  geom_point(size = 3) +
  geom_line(aes(x = as.numeric(rate),
                linetype = difficulty)) +
  facet_wrap(~ type, nrow = 1) +
  theme_bw() +
  labs(x = NULL,
       y = "Aggregated Percent Words Correct",
       linetype = NULL,
       shape = NULL) +
  scale_shape_manual(values = c(15, 19)) +
  scale_linetype_manual(values = c("solid", "longdash")) +
  theme(legend.position = c(1, 0),
        legend.justification = c(1.05, -0.1),
        legend.background = element_rect(color = "black"),
        legend.key.width = unit(1.5, "cm"))
```

Estimated Marginal Means plots between rate and lexical difficulty

## Post Hoc t-Tests

### Pooled Standard Deviation

```
data_agg %>% 
  dplyr::filter(!is.na(per_correct)) %>% 
  dplyr::group_by(rate, type, difficulty) %>% 
  dplyr::summarise(n = n(),
                   sd = sd(per_correct),
                   var = sd^2)
```

```
sd_pooled <- data_agg %>% 
  dplyr::group_by(rate, type, difficulty) %>% 
  dplyr::summarise(var = sd(per_correct)^2) %>% 
  dplyr::ungroup() %>% 
  dplyr::pull(var) %>% 
  mean() %>% 
  sqrt()

sd_pooled
```

```
[1] 9.985219
```

### Estimated Marginal Means

Note: design is perfectly balanced for difficulty, rate, and type.

```
fit_rmaov_3way %>% 
  emmeans::emmeans(~ difficulty*rate*type)
```

```
 difficulty          rate    type                emmean   SE  df lower.CL
 Lexically.Easy      X2.5.Hz Monosyllabic.Words    84.3 1.22 446     81.9
 Lexically.Difficult X2.5.Hz Monosyllabic.Words    71.0 1.22 446     68.6
 Lexically.Easy      X5.Hz   Monosyllabic.Words    81.0 1.22 446     78.6
 Lexically.Difficult X5.Hz   Monosyllabic.Words    67.7 1.22 446     65.3
 Lexically.Easy      X2.5.Hz Multisyllabic.Words   95.4 1.22 446     93.0
 Lexically.Difficult X2.5.Hz Multisyllabic.Words   74.5 1.22 446     72.1
 Lexically.Easy      X5.Hz   Multisyllabic.Words   86.7 1.22 446     84.3
 Lexically.Difficult X5.Hz   Multisyllabic.Words   81.3 1.22 446     78.9
 upper.CL
     86.7
     73.4
     83.4
     70.1
     97.8
     76.9
     89.1
     83.7

Warning: EMMs are biased unless design is perfectly balanced 
Confidence level used: 0.95
```

### Contrasts: difficulty X rate, within type of word

Correction for Multiple Comparisons (post hoc): Bonferroni’s Method

```
fit_cont <- fit_rmaov_3way %>% 
  emmeans::emmeans(~ difficulty*rate*type) %>% 
  emmeans::contrast(list("Mono: Interaction"  = c(1, -1, -1,  1, 
                                                  0,  0,  0,  0),
                         "Mono: 2.5 vs. 5"    = c(1, 1, -1, -1,
                                                  0, 0,  0,  0),
                         "Mono: Difficulty"   = c(1, -1, 1, -1,
                                                  0,  0, 0,  0),
                         "Multi: Interaction" = c(0,  0,  0,  0,
                                                  1, -1, -1,  1),
                         "Multi: 2.5Hz, diff"  = c(0, 0, 0, 0, 
                                                   1, -1, 0, 0),
                         "Multi: 5Hz, diff"  = c(0, 0, 0, 0, 
                                                 0, 0, 1, -1)),
                    adjust = "bon")
fit_cont
```

```
 contrast           estimate   SE  df t.ratio p.value
 Mono: Interaction   -0.0746 2.19 131 -0.034  1.0000 
 Mono: 2.5 vs. 5      6.6418 2.10 131  3.169  0.0114 
 Mono: Difficulty    26.6418 2.44 130 10.910  <.0001 
 Multi: Interaction  15.5224 2.19 131  7.074  <.0001 
 Multi: 2.5Hz, diff  20.8955 1.64 258 12.730  <.0001 
 Multi: 5Hz, diff     5.3731 1.64 258  3.273  0.0072 

P value adjustment: bonferroni method for 6 tests
```

```
fit_cont %>% 
  data.frame() %>% 
  dplyr::filter(contrast %in%
                  c("Mono: 2.5 vs. 5",
                    "Mono: Difficulty",
                    "Multi: 2.5Hz, diff",
                    "Multi: 5Hz, diff")) %>% 
  dplyr::mutate(cohen_d = estimate/sd_pooled)
```

# GLMM: Generalized (Logistic) Linear Mixed Effects Regression

Levels: Three

- Level 3 units = participants
  - IV’s (predictors, X)
    - age, years
    - working memory
    - long term memory
    - vocabulary
    - attention
- Level 2 units = sets of words
  - IV’s (predictors, X)
    - Rate: 2.5 vs. 5 Hz
    - Type: mono vs. multi syllabalic
    - Difficulty: Easy vs. Hard
- LEvel 1 units = individual words
  - DV (outcome, Y)
    - Correct: 0 = no, 1 = yes

---

## Null Model

```
fit_glmer_0 <- lme4::glmer(w_score ~ 1 + (1|id) + (1|words),
                           data = data_long,
                           family = binomial(link = "logit"))

summary(fit_glmer_0)
```

```
Generalized linear mixed model fit by maximum likelihood (Laplace
  Approximation) [glmerMod]
 Family: binomial  ( logit )
Formula: w_score ~ 1 + (1 | id) + (1 | words)
   Data: data_long

     AIC      BIC   logLik deviance df.resid 
    5954     5975    -2974     5948     8037 

Scaled residuals: 
    Min      1Q  Median      3Q     Max 
-7.7909  0.0969  0.2149  0.4078  3.3189 

Random effects:
 Groups Name        Variance Std.Dev.
 words  (Intercept) 3.6855   1.920   
 id     (Intercept) 0.1513   0.389   
Number of obs: 8040, groups:  words, 120; id, 67

Fixed effects:
            Estimate Std. Error z value Pr(>|z|)    
(Intercept)   2.0630     0.1893    10.9   <2e-16 ***
---
Signif. codes:  0 '***' 0.001 '**' 0.01 '*' 0.05 '.' 0.1 ' ' 1
```

### Variance Components

```
lme4::VarCorr(fit_glmer_0) %>% 
  print(comp = c("Variance","Std.Dev."))
```

```
 Groups Name        Variance Std.Dev.
 words  (Intercept) 3.68547  1.91976 
 id     (Intercept) 0.15131  0.38898
```

### ICC: intraclass-correlation

```
performance::icc(fit_glmer_0)
```

```
# Intraclass Correlation Coefficient

     Adjusted ICC: 0.538
  Conditional ICC: 0.538
```

## Investigate 3-way Interaction: Rate x Type x Difficulty

### Fit Nested Models

1. Main Effects Only
2. Interaction

```
fit_glmer_1 <- lme4::glmer(w_score ~ rate + type + difficulty +
                             (1|id) + (1|words),
                           data = data_long,
                           family = binomial(link = "logit"))

fit_glmer_2 <- lme4::glmer(w_score ~ rate*type*difficulty +
                             (1|id) + (1|words),
                           data = data_long,
                           control = glmerControl(optimizer = "optimx",
                                                  optCtrl = list(method = "nlminb")),
                           family = binomial(link = "logit"))
```

### Likelihood Ratio Tests: Compare Nested Models

More reliable than Wald-type tests of parameter estimates

```
anova(fit_glmer_0, fit_glmer_1, fit_glmer_2)
```

### Table of Parameter Estimates

```
texreg::screenreg(list(fit_glmer_1, fit_glmer_2))
```

```
==========================================================================================
                                                                Model 1       Model 2     
------------------------------------------------------------------------------------------
(Intercept)                                                         2.48 ***      2.44 ***
                                                                   (0.33)        (0.42)   
rate5 Hz                                                           -0.13         -0.03    
                                                                   (0.34)        (0.60)   
typeMultisyllabic Words                                             0.53          1.01    
                                                                   (0.37)        (0.73)   
difficultyLexically Difficult                                      -1.08 **      -0.99    
                                                                   (0.34)        (0.59)   
rate5 Hz:typeMultisyllabic Words                                                 -0.98    
                                                                                 (1.03)   
rate5 Hz:difficultyLexically Difficult                                           -0.21    
                                                                                 (0.83)   
typeMultisyllabic Words:difficultyLexically Difficult                            -0.98    
                                                                                 (1.03)   
rate5 Hz:typeMultisyllabic Words:difficultyLexically Difficult                    2.03    
                                                                                 (1.45)   
------------------------------------------------------------------------------------------
AIC                                                              5948.61       5954.23    
BIC                                                              5990.57       6024.15    
Log Likelihood                                                  -2968.31      -2967.11    
Num. obs.                                                        8040          8040       
Num. groups: words                                                120           120       
Num. groups: id                                                    67            67       
Var: words (Intercept)                                              3.26          3.20    
Var: id (Intercept)                                                 0.15          0.15    
==========================================================================================
*** p < 0.001; ** p < 0.01; * p < 0.05
```

```
fit_glmer_1 <- lme4::glmer(w_score ~ rate + ageyears+ type + difficulty+
                                    (1|id) + (1|words),
                          data = data_long %>% 
                            dplyr::filter(complete.cases(zmean_wm, 
                                                         zmean_attn, 
                                                         zmean_ltm, 
                                                         zmean_vocab)),
                           family = binomial(link = "logit"))


fit_glmer_2 <- lme4::glmer(w_score ~  rate + ageyears+ type+ difficulty+
                             zmean_wm  + zmean_attn +zmean_ltm+zmean_vocab+
                                    (1|id) + (1|words),
                           data = data_long  %>% 
                            dplyr::filter(complete.cases(zmean_wm, 
                                                         zmean_attn, 
                                                         zmean_ltm, 
                                                         zmean_vocab)),
                           control = glmerControl(optimizer = "optimx",
                                                  optCtrl = list(method = "nlminb")),
                           family = binomial(link = "logit"))
```

```
anova(fit_glmer_1, fit_glmer_2)
```

```
summary(fit_glmer_2)
```

```
Generalized linear mixed model fit by maximum likelihood (Laplace
  Approximation) [glmerMod]
 Family: binomial  ( logit )
Formula: 
w_score ~ rate + ageyears + type + difficulty + zmean_wm + zmean_attn +  
    zmean_ltm + zmean_vocab + (1 | id) + (1 | words)
   Data: data_long %>% dplyr::filter(complete.cases(zmean_wm, zmean_attn,  
    zmean_ltm, zmean_vocab))
Control: glmerControl(optimizer = "optimx", optCtrl = list(method = "nlminb"))

     AIC      BIC   logLik deviance df.resid 
  5821.7   5898.4  -2899.8   5799.7     7909 

Scaled residuals: 
    Min      1Q  Median      3Q     Max 
-8.0394  0.1006  0.2109  0.4078  3.5317 

Random effects:
 Groups Name        Variance Std.Dev.
 words  (Intercept) 3.26413  1.807   
 id     (Intercept) 0.06762  0.260   
Number of obs: 7920, groups:  words, 120; id, 66

Fixed effects:
                              Estimate Std. Error z value Pr(>|z|)   
(Intercept)                    1.70331    0.57905   2.942  0.00327 **
rate5 Hz                      -0.14914    0.34444  -0.433  0.66503   
ageyears                       0.08065    0.04864   1.658  0.09728 . 
typeMultisyllabic Words        0.51824    0.36612   1.415  0.15693   
difficultyLexically Difficult -1.06344    0.34458  -3.086  0.00203 **
zmean_wm                       0.06746    0.06841   0.986  0.32405   
zmean_attn                     0.11632    0.07002   1.661  0.09667 . 
zmean_ltm                     -0.00421    0.07551  -0.056  0.95554   
zmean_vocab                    0.13312    0.07560   1.761  0.07826 . 
---
Signif. codes:  0 '***' 0.001 '**' 0.01 '*' 0.05 '.' 0.1 ' ' 1

Correlation of Fixed Effects:
            (Intr) rat5Hz ageyrs typMlW dffcLD zmn_wm zmn_tt zmn_lt
rate5 Hz    -0.297                                                 
ageyears    -0.826  0.000                                          
typMltsyllW -0.211 -0.003  0.001                                   
dffcltyLxcD -0.303  0.000 -0.001  0.005                            
zmean_wm    -0.112  0.000  0.137  0.000 -0.001                     
zmean_attn  -0.029  0.000  0.036  0.001 -0.002 -0.277              
zmean_ltm    0.008  0.000 -0.009  0.000  0.000 -0.268 -0.134       
zmean_vocab  0.501  0.000 -0.601  0.001 -0.002 -0.239 -0.145 -0.258
```

```
fit_glmer_3 <- lme4::glmer(w_score ~ rate + ageyears+ type + difficulty+
                             zmean_wm + zmean_vocab+
                                    (1|id) + (1|words),
                           data = data_long  %>% 
                            dplyr::filter(complete.cases(zmean_wm, zmean_attn, zmean_ltm, zmean_vocab)),
                           control = glmerControl(optimizer = "optimx",
                                                  optCtrl = list(method = "nlminb")),
                           family = binomial(link = "logit"))
```

```
anova(fit_glmer_2, fit_glmer_3)
```

```
summary(fit_glmer_3)
```

```
Generalized linear mixed model fit by maximum likelihood (Laplace
  Approximation) [glmerMod]
 Family: binomial  ( logit )
Formula: 
w_score ~ rate + ageyears + type + difficulty + zmean_wm + zmean_vocab +  
    (1 | id) + (1 | words)
   Data: data_long %>% dplyr::filter(complete.cases(zmean_wm, zmean_attn,  
    zmean_ltm, zmean_vocab))
Control: glmerControl(optimizer = "optimx", optCtrl = list(method = "nlminb"))

     AIC      BIC   logLik deviance df.resid 
  5820.4   5883.2  -2901.2   5802.4     7911 

Scaled residuals: 
    Min      1Q  Median      3Q     Max 
-7.8683  0.1001  0.2105  0.4082  3.5426 

Random effects:
 Groups Name        Variance Std.Dev.
 words  (Intercept) 3.26429  1.8067  
 id     (Intercept) 0.07389  0.2718  
Number of obs: 7920, groups:  words, 120; id, 66

Fixed effects:
                              Estimate Std. Error z value Pr(>|z|)   
(Intercept)                    1.73083    0.58760   2.946  0.00322 **
rate5 Hz                      -0.14915    0.34447  -0.433  0.66504   
ageyears                       0.07807    0.04967   1.572  0.11599   
typeMultisyllabic Words        0.51825    0.36616   1.415  0.15696   
difficultyLexically Difficult -1.06346    0.34462  -3.086  0.00203 **
zmean_wm                       0.10287    0.06364   1.616  0.10603   
zmean_vocab                    0.15470    0.07329   2.111  0.03480 * 
---
Signif. codes:  0 '***' 0.001 '**' 0.01 '*' 0.05 '.' 0.1 ' ' 1

Correlation of Fixed Effects:
            (Intr) rat5Hz ageyrs typMlW dffcLD zmn_wm
rate5 Hz    -0.293                                   
ageyears    -0.831  0.000                            
typMltsyllW -0.208 -0.002  0.001                     
dffcltyLxcD -0.299  0.000 -0.001  0.005              
zmean_wm    -0.131  0.000  0.161  0.001 -0.002       
zmean_vocab  0.528  0.000 -0.630  0.001 -0.002 -0.423
```

```
fit_glmer_4 <- lme4::glmer(w_score ~ type + ageyears + rate*difficulty+ zmean_wm+ zmean_vocab+
                                    (1|id) + (1|words),
                           data = data_long  %>% 
                            dplyr::filter(complete.cases(zmean_wm, zmean_attn, zmean_ltm, zmean_vocab)),
                           control = glmerControl(optimizer = "optimx",
                                                  optCtrl = list(method = "nlminb")),
                           family = binomial(link = "logit"))
```

```
anova(fit_glmer_3,fit_glmer_4)
```

```
summary(fit_glmer_4)
```

```
Generalized linear mixed model fit by maximum likelihood (Laplace
  Approximation) [glmerMod]
 Family: binomial  ( logit )
Formula: 
w_score ~ type + ageyears + rate * difficulty + zmean_wm + zmean_vocab +  
    (1 | id) + (1 | words)
   Data: data_long %>% dplyr::filter(complete.cases(zmean_wm, zmean_attn,  
    zmean_ltm, zmean_vocab))
Control: glmerControl(optimizer = "optimx", optCtrl = list(method = "nlminb"))

     AIC      BIC   logLik deviance df.resid 
  5821.9   5891.7  -2901.0   5801.9     7910 

Scaled residuals: 
    Min      1Q  Median      3Q     Max 
-7.7802  0.1007  0.2110  0.4076  3.5330 

Random effects:
 Groups Name        Variance Std.Dev.
 words  (Intercept) 3.24846  1.8023  
 id     (Intercept) 0.07389  0.2718  
Number of obs: 7920, groups:  words, 120; id, 66

Fixed effects:
                                       Estimate Std. Error z value Pr(>|z|)   
(Intercept)                             1.84844    0.61255   3.018  0.00255 **
typeMultisyllabic Words                 0.52152    0.36534   1.427  0.15344   
ageyears                                0.07807    0.04966   1.572  0.11597   
rate5 Hz                               -0.38716    0.49032  -0.790  0.42976   
difficultyLexically Difficult          -1.29689    0.48698  -2.663  0.00774 **
zmean_wm                                0.10287    0.06364   1.616  0.10602   
zmean_vocab                             0.15469    0.07329   2.111  0.03480 * 
rate5 Hz:difficultyLexically Difficult  0.46583    0.68743   0.678  0.49800   
---
Signif. codes:  0 '***' 0.001 '**' 0.01 '*' 0.05 '.' 0.1 ' ' 1

Correlation of Fixed Effects:
            (Intr) typMlW ageyrs rat5Hz dffcLD zmn_wm zmn_vc
typMltsyllW -0.196                                          
ageyears    -0.797  0.001                                   
rate5 Hz    -0.400 -0.009  0.000                            
dffcltyLxcD -0.404 -0.004 -0.001  0.506                     
zmean_wm    -0.126  0.001  0.161  0.000 -0.001              
zmean_vocab  0.507  0.001 -0.630 -0.001 -0.002 -0.423       
rt5Hz:dffLD  0.285  0.010  0.000 -0.713 -0.708  0.000  0.000
```

```
fit_glmer_3r <- lme4::glmer(w_score ~ type + ageyears + rate + difficulty + zmean_wm+ zmean_vocab+
                                    (1|id) + (1|words),
                           data = data_long  %>% 
                            dplyr::filter(complete.cases(zmean_wm, zmean_vocab)),
                           control = glmerControl(optimizer = "optimx",
                                                  optCtrl = list(method = "nlminb")),
                           family = binomial(link = "logit"))


fit_glmer_5 <- lme4::glmer(w_score ~ type + ageyears + rate + difficulty*zmean_wm+ zmean_vocab+
                                    (1|id) + (1|words),
                           data = data_long  %>% 
                            dplyr::filter(complete.cases(zmean_wm, zmean_vocab)),
                           control = glmerControl(optimizer = "optimx",
                                                  optCtrl = list(method = "nlminb")),
                           family = binomial(link = "logit"))
```

```
anova(fit_glmer_3r,fit_glmer_5)
```

```
summary(fit_glmer_5)
```

```
Generalized linear mixed model fit by maximum likelihood (Laplace
  Approximation) [glmerMod]
 Family: binomial  ( logit )
Formula: 
w_score ~ type + ageyears + rate + difficulty * zmean_wm + zmean_vocab +  
    (1 | id) + (1 | words)
   Data: data_long %>% dplyr::filter(complete.cases(zmean_wm, zmean_vocab))
Control: glmerControl(optimizer = "optimx", optCtrl = list(method = "nlminb"))

     AIC      BIC   logLik deviance df.resid 
  5816.9   5886.7  -2898.4   5796.9     7910 

Scaled residuals: 
    Min      1Q  Median      3Q     Max 
-8.1877  0.0993  0.2116  0.4065  3.3387 

Random effects:
 Groups Name        Variance Std.Dev.
 words  (Intercept) 3.26711  1.8075  
 id     (Intercept) 0.07447  0.2729  
Number of obs: 7920, groups:  words, 120; id, 66

Fixed effects:
                                       Estimate Std. Error z value Pr(>|z|)   
(Intercept)                             1.73782    0.58831   2.954  0.00314 **
typeMultisyllabic Words                 0.51925    0.36621   1.418  0.15621   
ageyears                                0.07871    0.04976   1.582  0.11369   
rate5 Hz                               -0.15011    0.34456  -0.436  0.66310   
difficultyLexically Difficult          -1.08001    0.34477  -3.133  0.00173 **
zmean_wm                                0.21207    0.07800   2.719  0.00655 **
zmean_vocab                             0.15479    0.07345   2.108  0.03507 * 
difficultyLexically Difficult:zmean_wm -0.19195    0.08059  -2.382  0.01722 * 
---
Signif. codes:  0 '***' 0.001 '**' 0.01 '*' 0.05 '.' 0.1 ' ' 1

Correlation of Fixed Effects:
            (Intr) typMlW ageyrs rat5Hz dffcLD zmn_wm zmn_vc
typMltsyllW -0.207                                          
ageyears    -0.832  0.001                                   
rate5 Hz    -0.293 -0.003  0.000                            
dffcltyLxcD -0.299  0.005 -0.002  0.000                     
zmean_wm    -0.104  0.002  0.137 -0.001 -0.019              
zmean_vocab  0.529  0.001 -0.630  0.000 -0.002 -0.345       
dffcltyLD:_ -0.004 -0.002 -0.009  0.001  0.022 -0.577 -0.001
```

```
fit_glmer_6 <- lme4::glmer(w_score ~ type + ageyears + rate + 
                             difficulty*zmean_wm+ difficulty*zmean_vocab+
                                    (1|id) + (1|words),
                           data = data_long  %>% 
                            dplyr::filter(complete.cases(zmean_wm, zmean_vocab)),
                           control = glmerControl(optimizer = "optimx",
                                                  optCtrl = list(method = "nlminb")),
                           family = binomial(link = "logit"))
```

```
anova(fit_glmer_3r, fit_glmer_6)
```

```
summary(fit_glmer_6)
```

```
Generalized linear mixed model fit by maximum likelihood (Laplace
  Approximation) [glmerMod]
 Family: binomial  ( logit )
Formula: 
w_score ~ type + ageyears + rate + difficulty * zmean_wm + difficulty *  
    zmean_vocab + (1 | id) + (1 | words)
   Data: data_long %>% dplyr::filter(complete.cases(zmean_wm, zmean_vocab))
Control: glmerControl(optimizer = "optimx", optCtrl = list(method = "nlminb"))

     AIC      BIC   logLik deviance df.resid 
  5818.0   5894.7  -2898.0   5796.0     7909 

Scaled residuals: 
    Min      1Q  Median      3Q     Max 
-8.1469  0.1001  0.2110  0.4055  3.2936 

Random effects:
 Groups Name        Variance Std.Dev.
 words  (Intercept) 3.2664   1.8073  
 id     (Intercept) 0.0745   0.2729  
Number of obs: 7920, groups:  words, 120; id, 66

Fixed effects:
                                          Estimate Std. Error z value Pr(>|z|)
(Intercept)                                1.74360    0.58840   2.963  0.00304
typeMultisyllabic Words                    0.51949    0.36621   1.419  0.15603
ageyears                                   0.07872    0.04977   1.582  0.11372
rate5 Hz                                  -0.15054    0.34451  -0.437  0.66215
difficultyLexically Difficult             -1.08784    0.34486  -3.154  0.00161
zmean_wm                                   0.19126    0.08085   2.366  0.01799
zmean_vocab                                0.20208    0.08755   2.308  0.02099
difficultyLexically Difficult:zmean_wm    -0.15540    0.08870  -1.752  0.07978
difficultyLexically Difficult:zmean_vocab -0.08098    0.08193  -0.988  0.32294
                                            
(Intercept)                               **
typeMultisyllabic Words                     
ageyears                                    
rate5 Hz                                    
difficultyLexically Difficult             **
zmean_wm                                  * 
zmean_vocab                               * 
difficultyLexically Difficult:zmean_wm    . 
difficultyLexically Difficult:zmean_vocab   
---
Signif. codes:  0 '***' 0.001 '**' 0.01 '*' 0.05 '.' 0.1 ' ' 1

Correlation of Fixed Effects:
                          (Intr) typMlW ageyrs rat5Hz dffcLD zmn_wm zmn_vc
typMltsyllW               -0.207                                          
ageyears                  -0.832  0.001                                   
rate5 Hz                  -0.293 -0.002  0.000                            
dffcltyLxcD               -0.299  0.005 -0.002  0.000                     
zmean_wm                  -0.103  0.001  0.132 -0.001 -0.011              
zmean_vocab                0.450  0.001 -0.528 -0.001 -0.016 -0.419       
dffcltyLxcllyDffclt:zmn_w  0.000 -0.001 -0.007  0.001  0.010 -0.616  0.224
dffcltyLxcllyDffclt:zmn_v -0.010 -0.001 -0.001  0.001  0.024  0.258 -0.544
                          dffcltyLxcllyDffclt:zmn_w
typMltsyllW                                        
ageyears                                           
rate5 Hz                                           
dffcltyLxcD                                        
zmean_wm                                           
zmean_vocab                                        
dffcltyLxcllyDffclt:zmn_w                          
dffcltyLxcllyDffclt:zmn_v -0.416
```

```
fit_glmer_7 <- lme4::glmer(w_score ~ type + ageyears + rate + 
                             difficulty + zmean_wm+ difficulty*zmean_vocab+
                                    (1|id) + (1|words),
                           data = data_long  %>% 
                            dplyr::filter(complete.cases(zmean_wm, zmean_vocab)),
                           control = glmerControl(optimizer = "optimx",
                                                  optCtrl = list(method = "nlminb")),
                           family = binomial(link = "logit"))
```

```
anova(fit_glmer_3r, fit_glmer_7)
```

```
fit_glmer_5pure <- lme4::glmer(w_score ~ difficulty*zmean_wm+ zmean_vocab+
                                    (1|id) + (1|words),
                           data = data_long  %>% 
                            dplyr::filter(complete.cases(zmean_wm, zmean_vocab)),
                           control = glmerControl(optimizer = "optimx",
                                                  optCtrl = list(method = "nlminb")),
                           family = binomial(link = "logit"))

fit_glmer_8 <- lme4::glmer(w_score ~ difficulty*zmean_wm+ difficulty*zmean_vocab+
                                    (1|id) + (1|words),
                           data = data_long  %>% 
                            dplyr::filter(complete.cases(zmean_wm, zmean_vocab)),
                           control = glmerControl(optimizer = "optimx",
                                                  optCtrl = list(method = "nlminb")),
                           family = binomial(link = "logit"))
```

```
anova(fit_glmer_5, fit_glmer_5pure)
```

```
summary(fit_glmer_5pure)
```

```
Generalized linear mixed model fit by maximum likelihood (Laplace
  Approximation) [glmerMod]
 Family: binomial  ( logit )
Formula: w_score ~ difficulty * zmean_wm + zmean_vocab + (1 | id) + (1 |  
    words)
   Data: data_long %>% dplyr::filter(complete.cases(zmean_wm, zmean_vocab))
Control: glmerControl(optimizer = "optimx", optCtrl = list(method = "nlminb"))

     AIC      BIC   logLik deviance df.resid 
  5815.4   5864.3  -2900.7   5801.4     7913 

Scaled residuals: 
    Min      1Q  Median      3Q     Max 
-8.0816  0.0972  0.2122  0.4041  3.3017 

Random effects:
 Groups Name        Variance Std.Dev.
 words  (Intercept) 3.36714  1.8350  
 id     (Intercept) 0.07988  0.2826  
Number of obs: 7920, groups:  words, 120; id, 66

Fixed effects:
                                       Estimate Std. Error z value Pr(>|z|)    
(Intercept)                             2.61740    0.25309  10.342  < 2e-16 ***
difficultyLexically Difficult          -1.08495    0.34984  -3.101  0.00193 ** 
zmean_wm                                0.19540    0.07809   2.502  0.01234 *  
zmean_vocab                             0.22819    0.05804   3.932 8.44e-05 ***
difficultyLexically Difficult:zmean_wm -0.19097    0.08044  -2.374  0.01760 *  
---
Signif. codes:  0 '***' 0.001 '**' 0.01 '*' 0.05 '.' 0.1 ' ' 1

Correlation of Fixed Effects:
            (Intr) dffcLD zmn_wm zmn_vc
dffcltyLxcD -0.705                     
zmean_wm     0.024 -0.018              
zmean_vocab  0.014 -0.004 -0.339       
dffcltyLD:_ -0.027  0.022 -0.574 -0.009
```

```
texreg::screenreg(list(fit_glmer_5, 
                       fit_glmer_5pure, 
                       fit_glmer_8),
                  single.row = TRUE,
                  ci.test = 1)
```

```
=======================================================================================================
                                           Model 1             Model 2              Model 3            
-------------------------------------------------------------------------------------------------------
(Intercept)                                    1.74 (0.59) **      2.62 (0.25) ***      2.62 (0.25) ***
typeMultisyllabic Words                        0.52 (0.37)                                             
ageyears                                       0.08 (0.05)                                             
rate5 Hz                                      -0.15 (0.34)                                             
difficultyLexically Difficult                 -1.08 (0.34) **     -1.08 (0.35) **      -1.09 (0.35) ** 
zmean_wm                                       0.21 (0.08) **      0.20 (0.08) *        0.17 (0.08) *  
zmean_vocab                                    0.15 (0.07) *       0.23 (0.06) ***      0.28 (0.08) ***
difficultyLexically Difficult:zmean_wm        -0.19 (0.08) *      -0.19 (0.08) *       -0.15 (0.09)    
difficultyLexically Difficult:zmean_vocab                                              -0.08 (0.08)    
-------------------------------------------------------------------------------------------------------
AIC                                         5816.90             5815.45              5816.51           
BIC                                         5886.67             5864.29              5872.33           
Log Likelihood                             -2898.45            -2900.72             -2900.25           
Num. obs.                                   7920                7920                 7920              
Num. groups: words                           120                 120                  120              
Num. groups: id                               66                  66                   66              
Var: words (Intercept)                         3.27                3.37                 3.37           
Var: id (Intercept)                            0.07                0.08                 0.08           
=======================================================================================================
*** p < 0.001; ** p < 0.01; * p < 0.05
```

## Final Model

### Table of Parameter Estimates

```
texreg::screenreg(list(fit_glmer_5pure, 
                       extract_glmer_exp(fit_glmer_5pure,
                                         include.aic      = FALSE,
                                         include.bic      = FALSE,
                                         include.loglik   = FALSE,
                                         include.groups   = FALSE,
                                         include.nobs     = FALSE,
                                         include.variance = FALSE)),
                  custom.model.names = c("b(SE)", "OR [95 CI]"),
                  single.row = TRUE,
                  bold = TRUE,
                  ci.test = 1)
```

```
================================================================================
                                        b(SE)                OR [95 CI]         
--------------------------------------------------------------------------------
(Intercept)                                 2.62 (0.25) ***  13.70 [8.30; 22.61]
difficultyLexically Difficult              -1.08 (0.35) **    0.34 [0.17;  0.68]
zmean_wm                                    0.20 (0.08) *     1.22 [1.04;  1.42]
zmean_vocab                                 0.23 (0.06) ***   1.26 [1.12;  1.41]
difficultyLexically Difficult:zmean_wm     -0.19 (0.08) *     0.83 [0.70;  0.97]
--------------------------------------------------------------------------------
AIC                                      5815.45                                
BIC                                      5864.29                                
Log Likelihood                          -2900.72                                
Num. obs.                                7920                                   
Num. groups: words                        120                                   
Num. groups: id                            66                                   
Var: words (Intercept)                      3.37                                
Var: id (Intercept)                         0.08                                
================================================================================
*** p < 0.001; ** p < 0.01; * p < 0.05 (or Null hypothesis value outside the confidence interval).
```

Best Fit Model

```
texreg::screenreg(list(fit_glmer_5pure, 
                       extract_glmer_exp(fit_glmer_5pure,
                                         include.aic      = FALSE,
                                         include.bic      = FALSE,
                                         include.loglik   = FALSE,
                                         include.groups   = FALSE,
                                         include.nobs     = FALSE,
                                         include.variance = FALSE)),
                  custom.model.names = c("b(SE)", "OR [95 CI]"),
                  single.row = TRUE,
                  bold = TRUE,
                  ci.test = 1,
                 float.pos     = "hb",
                label         = "glmer_word_final",
                caption.above = TRUE,
               caption       = "Parameter Estimates: GLMM for words, Final Model")
```

================================================================================ b(SE) OR [95 CI]  
——————————————————————————– (Intercept) 2.62 (0.25) \*\*\* 13.70 [8.30; 22.61] difficultyLexically Difficult -1.08 (0.35) \*\* 0.34 [0.17; 0.68] zmean\_wm 0.20 (0.08) \* 1.22 [1.04; 1.42] zmean\_vocab 0.23 (0.06) \*\*\* 1.26 [1.12; 1.41] difficultyLexically Difficult:zmean\_wm -0.19 (0.08) \* 0.83 [0.70; 0.97] ——————————————————————————– AIC 5815.45  
BIC 5864.29  
Log Likelihood -2900.72  
Num. obs. 7920  
Num. groups: words 120  
Num. groups: id 66  
Var: words (Intercept) 3.37  
Var: id (Intercept) 0.08  
================================================================================ \*\*\* p < 0.001; \*\* p < 0.01; \* p < 0.05 (or Null hypothesis value outside the confidence interval).

### Interaction Plot

```
effects::Effect(focal.predictors = c("difficulty", "zmean_wm", "zmean_vocab"),
                mod = fit_glmer_5pure,
                xlevels = list(zmean_vocab = c(-1, 0, 1),
                               zmean_wm = seq(from = data_sub %>% 
                                                   dplyr::pull(zmean_wm) %>% 
                                                   min(na.rm = TRUE), 
                                                 to = data_sub %>% 
                                                   dplyr::pull(zmean_wm) %>% 
                                                   max(na.rm = TRUE), 
                                                 by = .1))) %>% 
  data.frame() %>% 
  dplyr::mutate(zmean_vocab = zmean_vocab %>% 
                  factor(labels = c(paste("Vocabulary, SD below Mean, z =", -1),
                                    paste("Vocabulary, Mean, z =", 0),
                                    paste("Vocabulary, SD above Mean, z =", 1)))) %>% 
  ggplot(aes(x = zmean_wm,
             y = fit,
             fill = difficulty,
             group = difficulty)) +
  geom_hline(yintercept = c(.75, 1),
             color = "gray",
             size = 1.5) +
  geom_ribbon(aes(ymin = fit - se,
                  ymax = fit + se),
              alpha = .3) +
  geom_line(aes(linetype = difficulty),
            size = 1) +
  facet_grid( ~ zmean_vocab) +
  theme_bw() +
  theme(legend.position = c(1, 0),
        legend.justification = c(1.05, -0.1),
        legend.key.width = unit(1.5, "cm"),
        legend.background = element_rect(color = "black")) +
  #scale_fill_manual(values = c("gray20", "gray60")) +
  scale_linetype_manual(values = c("solid", "longdash")) +
  labs(x = "Working Memory, Mean of z-Scores",
       y = "Predicted Probability of\nGetting a Word Correct",
       color = NULL,
       fill = NULL,
       linetype = NULL)
```

GLMM - Words: Marginal Probability of correct word recognition by WMC, controlling for vocabulary. Also illustrate the interaction between working memory and lexical difficulty

```
effects::Effect(focal.predictors = c("difficulty","zmean_wm", "zmean_vocab"),
                mod = fit_glmer_5pure,
                xlevels = list(zmean_wm = c(-1, 0, 1),
                               zmean_vocab = seq(from = data_sub %>% 
                                                   dplyr::pull(zmean_vocab) %>% 
                                                   min(na.rm = TRUE), 
                                                 to = data_sub %>% 
                                                   dplyr::pull(zmean_vocab) %>% 
                                                   max(na.rm = TRUE), 
                                                 by = .1))) %>% 
  data.frame() %>% 
  dplyr::mutate(zmean_wm = zmean_wm %>% 
                  factor(labels = c(paste("WMC, SD below Mean, z =", -1),
                                    paste("WMC, Mean, z =", 0),
                                    paste("WMC, SD above Mean, z =", 1)))) %>%  

  ggplot(aes(x = zmean_vocab,
             y = fit,
             fill = difficulty,
             group = difficulty)) +
  geom_hline(yintercept = c(.75, 1),
             color = "gray",
             size = 1.5) +
  geom_ribbon(aes(ymin = fit - se,
                  ymax = fit + se),
              alpha = .3) +
  geom_line(aes(linetype = difficulty),
            size = 1) +
  facet_grid( ~ zmean_wm) +
  theme_bw() +
  theme(legend.position = c(1, 0),
        legend.justification = c(1.05, -0.1),
        legend.key.width = unit(1.5, "cm"),
        legend.background = element_rect(color = "black")) +
  #scale_fill_manual(values = c("gray20", "gray60")) +
  scale_linetype_manual(values = c("solid", "longdash")) +
  labs(x = "Vocabulary, Mean of z-Scores",
       y = "Predicted Probability of\nGetting a Word Correct",
       color = NULL,
       fill = NULL,
       linetype = NULL)
```

GLMM - Words: Marginal Probability of correct word recognition by vocabulary, controlling for WMC

# Data Analysis for Sentences

## Import Data

### Experimental Data

```
data_exp_sent <- readxl::read_excel("ISP_KeywordScoring.xlsx",
                               range = "A1:F33232") %>% 
  dplyr::rename_all(tolower) %>% 
  dplyr::filter(id != "361") %>%                  # id = 361 has no experimental data
  dplyr::filter(condition == "Noise") %>%         
   dplyr::mutate(rate = rate %>% 
                  factor(levels = c("2.5", "5"),
                         labels = c("2.5 Hz", "5 Hz"))) %>% 
  dplyr::mutate_at(vars(id, rate, condition, sentences, keywords),
                   factor) 

  
tibble::glimpse(data_exp_sent)
```

```
Rows: 16,368
Columns: 6
$ id        <fct> 300, 300, 300, 300, 300, 300, 300, 300, 300, 300, 300, 30...
$ rate      <fct> 2.5 Hz, 2.5 Hz, 2.5 Hz, 2.5 Hz, 2.5 Hz, 2.5 Hz, 2.5 Hz, 2...
$ condition <fct> Noise, Noise, Noise, Noise, Noise, Noise, Noise, Noise, N...
$ sentences <fct> 7a1, 7a1, 7a1, 7a1, 7a2, 7a2, 7a2, 7a3, 7a3, 7a3, 7a4, 7a...
$ keywords  <fct> 1, 2, 3, 4, 5, 6, 7, 8, 9, 10, 11, 12, 13, 14, 15, 16, 17...
$ score     <dbl> 0, 0, 0, 1, 1, 1, 1, 0, 1, 1, 0, 0, 0, 1, 1, 1, 1, 0, 0, ...
```

### Merge together

```
data_long_s <- data_sub %>% 
  dplyr::right_join(data_exp_sent, by = "id")

#tibble::glimpse(data_long_s)
```

### Aggregate Data

Sample Size: Sentences, n = 66 participants x 2 rates) = 132

```
data_agg_s <- data_long_s %>% 
  dplyr::group_by(id, 
                  agemonths, ageyears,
                  zmean_wm,
                  zmean_ltm,
                  zmean_vocab,
                  zmean_attn,
                  rate) %>% 
  dplyr::summarise(set_n = n(),
                   prop_correct = sum(score)/n()) %>% 
  dplyr::mutate(per_correct = 100 * prop_correct) %>% 
  dplyr::ungroup() %>% 
  dplyr::mutate(set = interaction(rate) %>% 
                  as.numeric()) %>% 
  dplyr::arrange(id, set) %>% 
  dplyr::select(id, set, set_n, everything())


#tibble::glimpse(data_agg_s)
```

# Exploratory Data Analysis

## Summary Statistics: Aggregated Outcome: Percent Correct by Interruption Rate

```
data_agg_s %>% 
  dplyr::group_by(rate) %>% 
  dplyr::summarise(mean = mean(per_correct),
                   se   = sd(per_correct)/sqrt(n())) %>% 
  dplyr::mutate(cell = paste0(mean %>% round(3) %>% format(nsmall = 3), 
                              "  (", 
                              se %>% round(3) %>% format(nsmall = 3), 
                                     ")")) %>% 
  dplyr::select(-mean, -se) %>% 
  tidyr::pivot_wider(names_from = rate,
                     values_from = cell) %>% 
  pander::pander(caption = "Percent Correct, by  Rate")
```

Percent Correct, by Rate # Paired t-test


| 2.5 Hz | 5 Hz |
| --- | --- |
| 78.250 (0.828) | 91.325 (0.517) |

```
data_agg_s %>%
dplyr::arrange(id, rate) %>%
t.test(per_correct  ~ rate,
data = .,
id = "subject",
paired = TRUE)
```

```
    Paired t-test

data:  per_correct by rate
t = -19.722, df = 65, p-value < 2.2e-16
alternative hypothesis: true difference in means is not equal to 0
95 percent confidence interval:
 -14.39828 -11.75030
sample estimates:
mean of the differences 
              -13.07429
```

## Paired t-test Effect size

```
data_agg_s %>%
dplyr::arrange(id, rate) %>%
  
lsr::cohensD(per_correct ~ rate,
data = .,
method = "paired")
```

```
[1] 2.427561
```

## Visualization of Aggregated Data

```
data_agg_s %>% 
  dplyr::group_by(id, rate) %>% 
  #dplyr::summarise(prop_correct = sum(prop_correct)/n()) %>% 
  ggplot(aes(x = rate,
             y = prop_correct)) +
  geom_boxplot() +
  theme_bw()+
  labs(x = "Rate",
       y = "Aggregated proportion keywords Correct",
       linetype = NULL,
       shape = NULL)
```

Means keyword percent correct by rate

```
data_agg_s %>% 
  dplyr::group_by(id, rate, zmean_vocab) %>% 
  #dplyr::summarise(prop_correct = sum(score)/n()) %>% 
  ggplot(aes(x = zmean_vocab,
             y = prop_correct)) +
  geom_point() +
  theme_bw() +
  facet_grid(~ rate) +
  geom_smooth(method = "lm",
              formula = y ~ x + I(x^2))+
   labs(x = "Vocabulary",
       y = "Proportion keywords Correct",
       linetype = NULL,
       shape = NULL)
```

Bivariate distibution between vocabulary and keywords correct

```
data_agg_s %>% 
  dplyr::group_by(id, rate, zmean_ltm) %>% 
  #dplyr::summarise(prop_correct = sum(score)/n()) %>% 
  ggplot(aes(x = zmean_ltm,
             y = prop_correct)) +
  geom_point() +
  theme_bw() +
  facet_grid(~ rate) +
  geom_smooth(method = "lm",
              formula = y ~ x + I(x^2))+
  labs(x = "LTM retrieval",
       y = " Proportion keywords Correct",
       linetype = NULL,
       shape = NULL)
```

Bivariate distibution between LTM retrieval and keywords correct

```
data_agg_s %>% 
  dplyr::group_by(id, rate, zmean_wm) %>% 
  #dplyr::summarise(prop_correct = sum(score)/n()) %>% 
  ggplot(aes(x = zmean_wm,
             y = prop_correct)) +
  geom_point() +
  theme_bw() +
  facet_grid(~ rate) +
  geom_smooth(method = "lm",
              formula = y ~ x + I(x^2))+
   labs(x = "WMC",
       y = " Proportion keywords Correct",
       linetype = NULL,
       shape = NULL)
```

Bivariate distibution between WMC and keywords correct

```
data_agg_s %>% 
  dplyr::group_by(id, rate, zmean_attn) %>% 
  #dplyr::summarise(prop_correct = sum(score)/n()) %>% 
  ggplot(aes(x = zmean_attn,
             y = prop_correct)) +
  geom_point() +
  theme_bw() +
  facet_grid(~ rate, scale = "free_y") +
  geom_smooth(method = "lm",
                formula = y ~ x + I(x^2))
```

# GLMM = Generalized (Logistic) Linear Mixed Effects Regression

```
fit_glmer_0 <- lme4::glmer(score ~ 1 + (1|id) + (1|sentences),
                           data = data_long_s,
                           family = binomial(link = "logit"))

fit_glmer_1 <- lme4::glmer(score ~ rate + ageyears +
                                    (1|id) + (1|sentences),
                          data = data_long_s,
                           family = binomial(link = "logit"))
```

### ICC: intraclass-correlation

```
lme4::VarCorr(fit_glmer_0)
```

```
 Groups    Name        Std.Dev.
 sentences (Intercept) 1.76413 
 id        (Intercept) 0.45846
```

```
performance::icc(fit_glmer_0)
```

```
# Intraclass Correlation Coefficient

     Adjusted ICC: 0.502
  Conditional ICC: 0.502
```

Likelihood ratio test to compare nested models

```
anova(fit_glmer_0, fit_glmer_1)
```

```
summary(fit_glmer_1)
```

```
Generalized linear mixed model fit by maximum likelihood (Laplace
  Approximation) [glmerMod]
 Family: binomial  ( logit )
Formula: score ~ rate + ageyears + (1 | id) + (1 | sentences)
   Data: data_long_s

     AIC      BIC   logLik deviance df.resid 
 10265.6  10304.1  -5127.8  10255.6    16363 

Scaled residuals: 
     Min       1Q   Median       3Q      Max 
-11.7541   0.0954   0.1812   0.3491   2.0675 

Random effects:
 Groups    Name        Variance Std.Dev.
 sentences (Intercept) 2.7280   1.6517  
 id        (Intercept) 0.1692   0.4113  
Number of obs: 16368, groups:  sentences, 80; id, 66

Fixed effects:
            Estimate Std. Error z value Pr(>|z|)    
(Intercept)  0.45448    0.51998   0.874 0.382090    
rate5 Hz     1.19709    0.37987   3.151 0.001625 ** 
ageyears     0.16172    0.04519   3.579 0.000345 ***
---
Signif. codes:  0 '***' 0.001 '**' 0.01 '*' 0.05 '.' 0.1 ' ' 1

Correlation of Fixed Effects:
         (Intr) rat5Hz
rate5 Hz -0.358       
ageyears -0.853  0.000
```

```
fit_glmer_2 <- lme4::glmer(score ~  rate + ageyears+
                             zmean_wm  + zmean_attn + zmean_ltm + zmean_vocab+
                                    (1|id) + (1|sentences),
                           data = data_long_s,
                          control = glmerControl(optimizer = "optimx",
                                            optCtrl = list(method = "nlminb")),
                           family = binomial(link = "logit"))


#remove  age, Working memory and attention
fit_glmer_3 <- lme4::glmer(score ~ rate +
                             zmean_ltm + zmean_vocab+
                                    (1|id) + (1|sentences),
                           data = data_long_s,
                           control = glmerControl(optimizer = "optimx",
                                          optCtrl = list(method = "nlminb")),
                           family = binomial(link = "logit"))


#rate  interactions
fit_glmer_4 <- lme4::glmer(score ~ rate *
                             zmean_ltm + zmean_vocab+
                                    (1|id) + (1|sentences),
                           data = data_long_s,
                           control = glmerControl(optimizer = "optimx",
                                          optCtrl = list(method = "nlminb")),
                           family = binomial(link = "logit"))

fit_glmer_5 <- lme4::glmer(score ~ rate *zmean_vocab+
                             zmean_ltm + 
                                    (1|id) + (1|sentences),
                           data = data_long_s,
                           control = glmerControl(optimizer = "optimx",
                                          optCtrl = list(method = "nlminb")),
                           family = binomial(link = "logit"))

fit_glmer_6 <- lme4::glmer(score ~ rate *zmean_vocab*
                             zmean_ltm + 
                                    (1|id) + (1|sentences),
                           data = data_long_s,
                           control = glmerControl(optimizer = "optimx",
                                          optCtrl = list(method = "nlminb")),
                           family = binomial(link = "logit"))


fit_glmer_7 <- lme4::glmer(score ~ rate *zmean_vocab+
                            rate*zmean_ltm + 
                                    (1|id) + (1|sentences),
                           data = data_long_s,
                           control = glmerControl(optimizer = "optimx",
                                          optCtrl = list(method = "nlminb")),
                           family = binomial(link = "logit"))
```

```
anova(fit_glmer_1, fit_glmer_2)
```

```
anova(fit_glmer_1, fit_glmer_3)
```

Model without WM and attention fits better

```
anova(fit_glmer_2, fit_glmer_3)
```

Model without rate and LTM interactions fits better

```
anova(fit_glmer_3, fit_glmer_4)
```

Model without rate and vocabulary interactions fits better

```
anova(fit_glmer_3, fit_glmer_5)
```

Model without 3 way interactions fits better

```
anova(fit_glmer_3, fit_glmer_6)
```

```
texreg::screenreg(list(fit_glmer_4, 
                       fit_glmer_5,
                       fit_glmer_7),single.row = TRUE,
                  ci.test = 1)
```

```
=========================================================================================
                            Model 1              Model 2              Model 3            
-----------------------------------------------------------------------------------------
(Intercept)                     2.05 (0.27) ***      2.05 (0.27) ***      2.05 (0.27) ***
rate5 Hz                        1.20 (0.38) **       1.21 (0.38) **       1.21 (0.38) ** 
zmean_ltm                       0.17 (0.08) *        0.17 (0.07) *        0.18 (0.08) *  
zmean_vocab                     0.24 (0.06) ***      0.23 (0.06) ***      0.22 (0.06) ***
rate5 Hz:zmean_ltm              0.01 (0.07)                              -0.01 (0.08)    
rate5 Hz:zmean_vocab                                 0.04 (0.06)          0.05 (0.06)    
-----------------------------------------------------------------------------------------
AIC                         10250.25             10249.75             10251.72           
BIC                         10304.18             10303.67             10313.35           
Log Likelihood              -5118.13             -5117.88             -5117.86           
Num. obs.                   16368                16368                16368              
Num. groups: sentences         80                   80                   80              
Num. groups: id                66                   66                   66              
Var: sentences (Intercept)      2.73                 2.73                 2.73           
Var: id (Intercept)             0.11                 0.11                 0.11           
=========================================================================================
*** p < 0.001; ** p < 0.01; * p < 0.05
```

```
texreg::screenreg(list(fit_glmer_2, 
                       fit_glmer_3),
                  single.row = TRUE,
                  ci.test = 1)
```

```
===================================================================
                            Model 1             Model 2            
-------------------------------------------------------------------
(Intercept)                     1.70 (0.57) **      2.05 (0.27) ***
rate5 Hz                        1.20 (0.38) **      1.20 (0.38) ** 
ageyears                        0.04 (0.05)                        
zmean_wm                        0.02 (0.07)                        
zmean_attn                      0.01 (0.07)                        
zmean_ltm                       0.16 (0.08) *       0.17 (0.07) *  
zmean_vocab                     0.20 (0.08) *       0.24 (0.06) ***
-------------------------------------------------------------------
AIC                         10253.72            10248.29           
BIC                         10323.05            10294.51           
Log Likelihood              -5117.86            -5118.15           
Num. obs.                   16368               16368              
Num. groups: sentences         80                  80              
Num. groups: id                66                  66              
Var: sentences (Intercept)      2.73                2.73           
Var: id (Intercept)             0.11                0.11           
===================================================================
*** p < 0.001; ** p < 0.01; * p < 0.05
```

### Final Model: Parameter Estimates

```
texreg::screenreg(list(fit_glmer_3, 
                       extract_glmer_exp(fit_glmer_3,
                                         include.aic      = FALSE,
                                         include.bic      = FALSE,
                                         include.loglik   = FALSE,
                                         include.groups   = FALSE,
                                         include.nobs     = FALSE,
                                         include.variance = FALSE)),
                  custom.model.names = c("b(SE)", "OR [95 CI]"),
                  single.row = TRUE,
                  ci.test = 1)
```

```
=====================================================================
                            b(SE)                OR [95 CI]          
---------------------------------------------------------------------
(Intercept)                     2.05 (0.27) ***  7.76 [4.54; 13.24] *
rate5 Hz                        1.20 (0.38) **   3.31 [1.56;  7.03] *
zmean_ltm                       0.17 (0.07) *    1.19 [1.03;  1.38] *
zmean_vocab                     0.24 (0.06) ***  1.27 [1.13;  1.43] *
---------------------------------------------------------------------
AIC                         10248.29                                 
BIC                         10294.51                                 
Log Likelihood              -5118.15                                 
Num. obs.                   16368                                    
Num. groups: sentences         80                                    
Num. groups: id                66                                    
Var: sentences (Intercept)      2.73                                 
Var: id (Intercept)             0.11                                 
=====================================================================
*** p < 0.001; ** p < 0.01; * p < 0.05 (or Null hypothesis value outside the confidence interval).
```

```
texreg::screenreg(list(fit_glmer_3, 
                       extract_glmer_exp(fit_glmer_3,
                                         include.aic      = FALSE,
                                         include.bic      = FALSE,
                                         include.loglik   = FALSE,
                                         include.groups   = FALSE,
                                         include.nobs     = FALSE,
                                         include.variance = FALSE)),
                  custom.model.names = c("b(SE)", "OR [95 CI]"),
                  single.row = TRUE,
                  ci.test = 1,
                  float.pos     = "hb",
                label         = "glmer_word_final",
                caption.above = TRUE,
               caption       = "Parameter Estimates: GLMM for keywords/sentence, Final Model")
```

===================================================================== b(SE) OR [95 CI]  
——————————————————————— (Intercept) 2.05 (0.27) \*\*\* 7.76 [4.54; 13.24]  *rate5 Hz 1.20 (0.38)  **3.31 [1.56; 7.03]  *zmean\_ltm 0.17 (0.07)*  1.19 [1.03; 1.38]  *zmean\_vocab 0.24 (0.06)***  1.27 [1.13; 1.43]*  ——————————————————————— AIC 10248.29  
BIC 10294.51  
Log Likelihood -5118.15  
Num. obs. 16368  
Num. groups: sentences 80  
Num. groups: id 66  
Var: sentences (Intercept) 2.73  
Var: id (Intercept) 0.11  
===================================================================== \*\*\* p < 0.001; \*\* p < 0.01; \* p < 0.05 (or Null hypothesis value outside the confidence interval).

```
data_sub %>% 
  dplyr::select(zmean_ltm, zmean_vocab) %>% 
  summary()
```

```
   zmean_ltm        zmean_vocab      
 Min.   :-2.1357   Min.   :-2.94286  
 1st Qu.:-0.3976   1st Qu.:-0.55692  
 Median : 0.1390   Median : 0.04076  
 Mean   : 0.0000   Mean   : 0.00000  
 3rd Qu.: 0.5143   3rd Qu.: 0.48878  
 Max.   : 1.4166   Max.   : 2.24508  
 NA's   :1         NA's   :1
```

## Model Plots

```
effects::Effect(focal.predictors = c("rate","zmean_ltm", "zmean_vocab"),
                mod = fit_glmer_3,
                xlevels = list(zmean_ltm = c(-1, 0, 1),
                               zmean_vocab = seq(from = data_sub %>% 
                                                   dplyr::pull(zmean_vocab) %>% 
                                                   min(na.rm = TRUE), 
                                                 to = data_sub %>% 
                                                   dplyr::pull(zmean_vocab) %>% 
                                                   max(na.rm = TRUE), 
                                                 by = .1))) %>% 
  data.frame() %>% 
  dplyr::mutate(zmean_ltm = zmean_ltm %>% 
                  factor(labels = c(paste("LTM, SD below Mean, z =", -1),
                                    paste("LTM, Mean, z =", 0),
                                    paste("LTM, SD above Mean, z =", 1)))) %>% 
                  
  
  ggplot(aes(x = zmean_vocab,
             y = fit,
             fill = rate,
             group = rate)) +
  geom_hline(yintercept = c(.75, 1),
             color = "gray",
             size = 1.5) +
  geom_ribbon(aes(ymin = fit - se,
                  ymax = fit + se),
              alpha = .3) +
  geom_line(aes(linetype = rate),
            size = 1) +
  facet_grid( ~ zmean_ltm) +
  theme_bw() +
  theme(legend.position = c(1, 0),
        legend.justification = c(1.05, -0.1),
        legend.key.width = unit(1.5, "cm"),
        legend.background = element_rect(color = "black")) +
  #scale_fill_manual(values = c("gray20", "gray60")) +
  scale_linetype_manual(values = c("solid", "longdash")) +
  labs(x = "Vocabulary, Mean of z-Scores",
       y = "Predicted Probability of\nGetting a Keyword Correct",
       color = "rate",
       fill = "Rate",
       linetype = "Rate")
```

GLMM - Sentences: Marginal Probability of correct keyword recognition by vocabulary, controlling for LTM retrieval

```
effects::Effect(focal.predictors = c("rate","zmean_ltm", "zmean_vocab"),
                mod = fit_glmer_3,
                xlevels = list(zmean_vocab = c(-1, 0, 1),
                               zmean_ltm = seq(from = data_sub %>% 
                                                   dplyr::pull(zmean_vocab) %>% 
                                                   min(na.rm = TRUE), 
                                                 to = data_sub %>% 
                                                   dplyr::pull(zmean_vocab) %>% 
                                                   max(na.rm = TRUE), 
                                                 by = .1))) %>% 
  data.frame() %>% 
  dplyr::mutate(zmean_vocab = zmean_vocab %>% 
                  factor(labels = c(paste("Vocabulary, SD below Mean, z =", -1),
                                    paste("Vocabulary, Mean, z =", 0),
                                    paste("Vocabulary, SD above Mean, z =", 1)))) %>%  
  
               #   forcats::fct_rev()) %>% 
  
  
  ggplot(aes(x = zmean_ltm,
             y = fit,
             fill = rate,
             group = rate)) +
  geom_hline(yintercept = c(.75, 1),
             color = "gray",
             size = 1.5) +
  geom_ribbon(aes(ymin = fit - se,
                  ymax = fit + se),
              alpha = .3) +
  geom_line(aes(linetype = rate),
            size = 1) +
  facet_grid( ~ zmean_vocab) +
  theme_bw() +
  theme(legend.position = c(1, 0),
        legend.justification = c(1.05, -0.1),
        legend.key.width = unit(1.5, "cm"),
        legend.background = element_rect(color = "black")) +
  #scale_fill_manual(values = c("gray20", "gray60")) +
  scale_linetype_manual(values = c("solid", "longdash")) +
  labs(x = "LTM retrieval, Mean of z-Scores",
       y = "Predicted Probability of\nGetting a Keyword Correct",
       color = "rate",
       fill = "Rate",
       linetype = "Rate")
```

GLMM - Sentences: Marginal Probability of correct keyword recognition by LTM retrieval, controlling for vocabulary
